# Gallic Acid Ameliorated Impaired Lipid Homeostasis in a Mouse Model of High-Fat Diet—and Streptozotocin-Induced NAFLD and Diabetes through Improvement of *β*-oxidation and Ketogenesis

**DOI:** 10.3389/fphar.2020.606759

**Published:** 2021-02-12

**Authors:** Jung Chao, Hao-Yuan Cheng, Ming-Ling Chang, Shyh-Shyun Huang, Jiunn-Wang Liao, Yung-Chi Cheng, Wen-Huang Peng, Li-Heng Pao

**Affiliations:** ^1^ Department of Chinese Pharmaceutical Sciences and Chinese Medicine Resources, Chinese Medicine Research Center, China Medical University, Taichung, Taiwan; ^2^ Department of Nursing, Chung-Jen Junior College of Nursing, Health Sciences and Management, Chia-Yi, Taiwan; ^3^ Division of Hepatology, Department of Gastroenterology and Hepatology, Liver Research Center, Chang Gung Memorial Hospital, Linko, Taiwan; ^4^ School of Pharmacy, China Medical University, Taichung, Taiwan; ^5^ Graduate Institute of Veterinary Pathology, National Chung Hsing University, Taichung, Taiwan; ^6^ Department of Pharmacology, Yale University School of Medicine, New Haven, CT, United States; ^7^ Department of Chinese Pharmaceutical Sciences and Chinese Medicine Resources, China Medical University, Taichung, Taiwan; ^8^ Graduate Institute of Health Industry Technology, Research Center for Food and Cosmetic Safety, and Research Center for Chinese Herbal Medicine, College of Human Ecology, Chang Gung University of Science and Technology, Taoyuan, Taiwan; ^9^ Department of Gastroenterology and Hepatology, Chang Gung Memorial Hospital, Linko, Taiwan

**Keywords:** gallic acid, non-alcoholic fatty liver disease, metabolomics, diabetes, *β*-oxidation, ketogenesis

## Abstract

Gallic acid (GA) is a simple polyphenol found in food and traditional Chinese medicine. Here, we determined the effects of GA administration in a combined mouse model of high-fat diet (HFD)-induced obesity and low-dose streptozotocin (STZ)-induced hyperglycemia, which mimics the concurrent non-alcoholic fatty liver disease (NAFLD) and type 2 diabetes pathological condition. By combining the results of physiological assessments, pathological examinations, metabolomic studies of blood, urine, liver, and muscle, and measurements of gene expression, we attempted to elucidate the efficacy of GA and the underlying mechanism of action of GA in hyperglycemic and dyslipidemic mice. HFD and STZ induced severe diabetes, NAFLD, and other metabolic disorders in mice. However, the results of liver histopathology and serum biochemical examinations indicated that daily GA treatment alleviated the high blood glucose levels in the mice and decelerated the progression of NAFLD. In addition, our results show that the hepatoprotective effect of GA in diabetic mice occurs in part through a partially preventing disordered metabolic pathway related to glucose, lipids, amino acids, purines, and pyrimidines. Specifically, the mechanism responsible for alleviation of lipid accumulation is related to the upregulation of *β*-oxidation and ketogenesis. These findings indicate that GA alleviates metabolic diseases through novel mechanisms.

## Introduction

Diabetes is a multi-etiological chronic metabolic disorder that is primarily characterized by pathological and physiological changes induced by insulin resistance and impaired insulin secretion. Because of changes in lifestyle and improved living standards, the global incidence of diabetes has gradually increased from 153 million in 1980 to 347 million people in 2010 (Danaei et al., 2011). Moreover, the mortality rate associated with vascular complications induced by diabetes, including diabetic nephropathy, has also gradually increased. The World Health Organization estimated that by 2030, diabetes would become the seventh leading cause of death worldwide (Danaei et al., 2011). It is therefore imperative to develop safe and effective diabetic drugs as well as preventive and therapeutic strategies to combat diabetes-related secondary complications.

Naturally occurring phenolic acids are prevalent in human food products. Polyphenol compounds and polyphenol-rich diets facilitate prevention and treatment of diabetes (Dembinska-Kiec et al., 2008; Xiao et al. 2015). Among various types of naturally occurring polyphenols, GA is one of the simplest and exists in free forms, such as tannins, ellagitannin, theaflavin-3-gallate, and epigallocatechin gallate. GA is found in many dietary substances, including vegetables, fruits, red wine, and tea (Manach et al., 2005; Subramanian et al., 2015); Among them, tea is a particularly important source of GA. (Tomás-Barberán and Clifford, 2000). Many reports have been confirmed that catechin and gallic acid (GA) are the most important compounds among the other tea constituents (Liang et al., 2007; Wang et al., 2001; Mok et al., 2002; Lee et al., 2005; Yue et al., 2014). Previous research also pointed out that free-form GA also exists in tea (Kongpichitchoke et al., 2016). Different teas have different amounts of GA, ranging from 0.1% to 2% (Kongpichitchoke et al., 2016). It is worth noting that GA occurs in tea in free and esterified forms (Matthew et al., 1997). Esterified forms include gallocatechin gallate (GCG), catechin gallate (CG), epicatechin gallate (ECG), epigallocatechin gallate (EGCG) (Quan et al., 2011).

GA has been reported to facilitate favorable biological activities, including radical scavenging, oxidation inhibition (Hsu and Yen, 2007), inflammation alleviation (Hsiang et al., 2013), obesity reduction (Hsu and Yen, 2007; Oi et al., 2012), and tumor suppression (Subramanian et al., 2015). Moreover, GA functions to alleviate metabolic diseases, including NAFLD (Chao et al., 2014) and diabetes, by upregulating the peroxisome proliferation-activated receptor (PPAR) in the liver, muscles, and adipose tissue, thereby reducing blood glucose (Gandhi et al., 2014), hepatic lipid peroxidation (Punithavathi et al., 2011b), diabetes induced myocardial dysfunction (Patel and Goyal, 2011), and diabetic nephropathy (Ahad et al., 2015) while enhancing pancreatic antioxidase activity (Punithavathi et al., 2011a). Additionally, studies have reported that GA inhibits the activities of glycogen phosphorylase (Kyriakis et al., 2015) and α-glucosidase (Benalla et al., 2010). GA was also shown to protect pancreatic islet RINm5F *β*-cells from the proapoptotic effects of glucolipotoxicity (Sameermahmood et al., 2010). Hence, GA has been clearly shown to facilitate favorable activities that alleviate diabetic symptoms. As GA intake, theoretically, facilitates systematic protection of the human body, a holistic approach should be taken to analyze its effects.

Naturally occurring products or functional foods exert moderate effects on the human body, in contrast to chemical drugs that serve as specific inhibitors of selective targets and thus exert considerable effects. Therefore, analyzing a naturally occurring product from a transcriptome or proteome perspective cannot comprehensively reflect its true functional mechanisms. Alternatively, a systems biology perspective allows for identification of metabolites in the terminal of biological systems, along with corresponding minor changes in their gene and protein expressions (Taylor et al., 2002). Consequently, analyzing the changes in the metabolites of an organism facilitates identification of subtle changes in the organism.

Previous studies primarily used conventional blood biochemical markers and examination of histopathological sections to investigate the mechanisms by which GA alleviates metabolic diseases (Hsu and Yen, 2007; Punithavathi et al., 2011b; Oi et al., 2012; Gandhi et al., 2014; Ahad et al., 2015), thus examining from a protein target perspective alone (Kyriakis et al., 2105; Benalla et al., 2010), which is largely unilateral in its understanding of related functional mechanisms. Therefore, the functions and mechanisms of GA in alleviating metabolic diseases merit re-examination. In this study, we hypothesized, when attempting to elucidate the preventive effects and mechanisms of GA on diabetes and non-alcoholic fatty liver disease, using these results is likely to provide strong evidence to support the preventive effects of daily consumption of this functional food on metabolic diseases. This study aimed to investigate the beneficial effects of GA on diabetes and non-alcoholic steatohepatitis (NASH) by metabolomics using an animal model. We used a high-fat diet (HFD) and streptozotocin (STZ) to induce diabetes in an animal model so as to evaluate the pharmacodynamics and mechanisms of GA in alleviating diabetes and NASH. The HFD-induced NAFLD model used showed signs similar to the NAFLD pathogenesis experienced by humans, incorporating obesity and other metabolic complications (Hebbard and George, 2011). A typical model, used in pharmacology, is to damage pancreatic *β*-cells through STZ to realize insufficient insulin secretion, thus inducing hyperglycemia and diabetes (Szkudelski, 2001). This study used a composite induction model that integrated the features of the aforementioned two models, conforming to the pathogenesis of type 2 diabetes in humans (Mu et al., 2006; Sawant et al., 2006). Moreover, previous studies have reported that this model can successfully induce NASH (Lo et al., 2011). Therefore, the present study also used the model to evaluate the effect of GA on NASH. A previous study used an NMR-based metabolomics strategy to examine the effect of GA on mice with NAFLD (Chao et al., 2014) by analyzing the changes in serum and urinary metabolites. This strategy enabled the identification of total metabolic changes in various tissues and organs but failed to reflect organ-oriented metabolic changes; hence, the specificity of related biomarkers needs to be further verified. Furthermore, high blood glucose and insulin resistance induced by HFD and STZ can affect various organs and tissues in the human body, while the interactions between the organs can also augment disease progression. As such, here the changes in blood and urinary metabolites and the liver and muscular tissues metabolites were measured, the organs most likely to be affected by diabetes, to comprehensively investigate the functional mechanisms of GA in mice with diabetes and NASH. This study will therefore show results and proposed pathways that will help elucidate the multiple targets involved in the hepatoprotective activities of GA.

## Materials and Methods

### Chemicals

GA (98%), heavy water (D_2_O; 99.9%), chloroform-d containing tetramethylsilane (99.9%), STZ, 6-hydroxy-2,5,7,8-tetramethylchromane-2-carboxylic acid (Trolox), and potassium dihydrogen phosphate (KH_2_PO_4_) were purchased from Sigma-Aldrich (St. Louis, MO, USA). Trimethylsilane propionic acid sodium salt (TSP) was obtained from Merck (Darmstadt, Germany). Sodium deuteroxide (NaOD) was purchased from Cambridge Isotope Laboratories (Tewksbury, MA, USA). Test Diets 58Y1 (St. Louis, MO, USA) was used as the HFD, and LabDiet 5010 (Richmond, IN, USA) was used as the control diet.

### Animal Study and Sample Collection

In this study, the mice were raised according to the Guide for the Care and Use of Laboratory Animals published by the National Institutes of Health. The animal experimental protocol was approved by the Laboratory Animal Center Committee of Ghang Gung University of Science and Technology (IACUC-2014-009).

Supplementary Figure S1 illustrates the experimental design flowchart. The animal experiment was designed and revised according to the 2010 Amendment Draft on Assessment Methods of Blood Glucose Function Regulated by Health Food (Ministry of Health and Welfare) and the model described in a previous study (Mu et al., 2006). Male 8 week old C57BL6/J mice (20–25 g) were purchased from BioLASCO, Taiwan. To decrease the variance of the model, each mouse was independently raised in a separate cage, with constant temperature and relative humidity of 22 ± 1°C and 55% ± 5%, respectively. The light–dark cycle was 12 h (08:00 to 20:00).

Supplementary Table S1 describes the diet composition and caloric ratio for each group. No limit was set for the amount of the regular diet and HFD given to the mice. The normal group was fed the regular diet, while the remaining groups were fed the HFD (Test Diets 58Y1). After night weeks, the mice were weighed (Supplementary Figure S2). An average weight >40 g was set as the criterion for initiating the STZ injection. To prepare the injection reagent, STZ was dissolved in normal saline, and 0.1 M sodium citrate (pH = 4.5) was used as the buffer solution. Each injection dose was 40 mg/kg. STZ was injected intraperitoneally once per day for two consecutive days in 9th week. Before the first injection, the mice were fasted for 16 h. After one week of STZ administration, the blood glucose levels of the mice were measured, and the mice were divided into three groups: normal group (n = 8), diabetic group (HFD + STZ (n = 8), and treatment group (0.2% GA in HFD; n = 8) according to the average blood glucose level. For the treatment, 0.2% GA was mixed with the HFD. The food intake and weights of the mice were measured weekly.

The amount of GA administered to the mice was determined based on the daily intake of tannic acid by humans (Sanyal et al., 1997). Tannic acid is a derivative of GA. The International Agency of Research on Cancer reported that its average human intake in the United States is approximately 1 g (Sanyal et al., 1997). Therefore, according to the average weight (60 kg) and daily tannic acid intake (1 g) of human adults published by the US Food and Drug Administration, the daily average GA intake is approximately 16.67 mg/kg, and states that multiplying the human equivalent dose by 12.3 yields the animal dose for mice (Food and Drug Administration, 2005); hence, the daily GA intake for mice is approximately 205.41 mg/kg. Accordingly, in the present study, the daily HFD intake for each mouse was approximately 3.0 g, the average weight of the mice was 40 g, and the diet for the treatment group contained 0.2% GA; thus, the average daily GA intake of each mouse was 150 mg/kg.

The mice were randomly selected on the 10th, 11th, 12th, and 15 th week for blood glucose testing to evaluate the effect of GA on blood glucose levels in mice. The oral glucose tolerance test (OGTT) was conducted on the 16 th week to assess the glucose metabolism of the mice. Mouse urine samples were collected on the 17 th week between 18:00 and 00:00 according to the circadian rhythm of the mice. The urine samples were centrifuged at 13,200 rpm to acquire the supernatant, and then stored in a −80°C freezer. A urine sample was discarded if it was contaminated by mouse feces during sample collection.

The mice were fasted for 16 h before being euthanized by CO_2_ to ensure that liver glycogen was completely utilized. The blood samples of the euthanized mice were loaded into heparin-free or ethylenediaminetetraacetic acid-free tubes and incubated at 23–25°C for 30 min, followed by centrifugation (3000 rpm) at 4°C for 20 min. The supernatants recovered as the serum samples of the mice were used for conducting biochemical analysis. An organ weighing scale and the hematoxylin and eosin (H&E) staining method were used to assess the fat accumulation of the mice. Liver tissues were partially removed from the euthanized mice and immersed in the RNAlater® solution (Thermo Fisher Scientific Inc., Waltham, MA, USA). The tissue samples were stored at −20°C to prevent RNA degradation and for later analysis *via* quantitative polymerase chain reaction (qPCR). The blood, urine, liver, and muscular tissue samples were used to conduct metabolomics analysis.

### Serum Biochemical Analysis, Serum Insulin and Biological Activity Tests

The following serum biochemical markers were assessed using a dry serum biochemical analyzer (Dry-Chem 4000i, Fujifilm, Saitama, Japan): aspartate (AST) and alanine transaminases (ALT), high-density lipoprotein (HDL), triglycerides (TG), and total cholesterol (TCHO).

### OGTT

The OGTT was used to determine the diabetes severity in the mice. A glucose solution (2 g/kg) was prepared one day prior to the test and settled overnight. The mice were fasted for 16 h before the test. First, the fasting glucose test was conducted (0 min); subsequently, the blood samples were collected at 30, 60, 90, and 120 min after orally feeding the mice to measure their blood glucose level, followed by statistical analysis.

### Histopathological Analysis

After the mice were euthanized, the liver lobes, thigh scale muscle, and organ fat were acquired and immersed in 10% formaldehyde to create paraffin sections (thickness = 4 µm), followed by H&E staining. Accordingly, changes in the tissues were observed. The liver tissue sections were also dyed using Sirius red, a collagen fiber-specific dye, to determine liver fibrosis.

### NMR Metabolomics

The blood and urine samples frozen in the refrigerator were defrosted at room temperature (25°C), followed by centrifuging at 13,000 rpm for 15 min to remove insoluble substances. The blood samples were prepared by evenly mixing 100 µL of serum with 500 µL of phosphate buffer (0.57 g K_2_HPO_4_ + 0.0981 g NaH_2_PO_4_ + 0.81 NaCl in 100 ml D_2_O, pH = 7.4). The urine samples were prepared by evenly mixing 100 µL of urine with 500 µL of phosphate buffer (0.38143 g K_2_HPO_4_ + 0.06568 g NaH_2_PO_4_ + 0.81 NaCl in 100 mL D_2_O, pH = 7.4, 1 mM TSP). Next, 550 µL of the blood or urine sample was loaded into a 5 mm NMR tube. The liver and muscle samples were processed according to the method described previously (An et al., 2013; Chao et al., 2014). Liver tissue and muscle tissue samples (∼50 mg) were extracted with 0.4285 ml of pre-cooled methanol–water mixture (4/2.85, v/v) using a tissue homogenizer. After adding 0.4 ml chloroform to the methanol–water mixture, the solutions were separated into an upper methanol–water phase (with polar metabolites) and a lower chloroform phase (with lipophilic metabolites), which were collected separately following centrifugation (1,000 × g, 4°C, 10 min) and methanol–water or chloroform were then removed in vacuo. The polar extract was reconstituted using 600 µL of phosphate buffer (0.38143 g K_2_HPO_4_ + 0.06568 g NaH_2_PO_4_ + 0.81 NaCl in 100 ml D_2_O, pH = 7.4, 1 mM TSP). The lipophilic extract was reconstituted using 600 µL of chloroform-d containing TMS. Then, 550 µL of each sample was transferred to a 5 mm NMR tube for NMR analysis.

The NMR instrument in the High Field Nuclear Magnetic Resonance Center of Academia Sinica was used. The experimental conditions were configured according to previous literature (Chao et al., 2014; Beckonert et al., 2007). The spectrum analysis was conducted according to the method described previously (Supplementary Table S2) (Beckonert et al., 2007; Chao et al., 2014).

The resulting ^1^H-NMR spectra were manually phased, baseline corrected, and calibrated to TSP or TMS at *δ* = 0.00 ppm using Mestrenova software (version 8.0.2, Mestrelab research S.L.). The blood spectrum was not subjected to normalization. In case of urine samples, the glucose signals (3.220–3.275, 3.300–3.560, 3.695–3.925, 4.600–4.700, and 5.160–5.280 ppm) and urea signal (5.600–6.000 ppm) were removed from the spectrum, and it was pretreated using total area normalization. The liver and muscular tissue spectra were normalized according to the tissue wet weight values.

For spectral resonance assignment purposes, ^1^H-^1^H correlation spectroscopy (COSY), ^1^H-^1^H total correlation spectroscopy (TOCSY), ^1^H J-resolved spectroscopy (JRES), ^1^H-^13^C heteronuclear single-quantum coherence (HSQC), and heteronuclear multiple bond correlation (HMBC) 2D NMR spectra were acquired on selected samples and processed as previously reported (An et al., 2013).

The resulting NMR datasets were imported into SIMCA-P version 13.0 (Umetrics, Umea, Sweden). All variables were scaled to Pareto (par) for multivariate statistical analyses and analyzed according to the previous study (Chao et al., 2014).

All results are presented as means ± SE. The statistical analysis was performed using one-way analysis of variance (ANOVA), followed by Bonferroni’s post hoc test. The criterion used for statistical significance was *p* < 0.05.

### Real-Time Fluorescence qPCR Analysis

The liver tissue samples were immersed in the RNAlater® solution (Thermo Fisher Scientific Inc.) and stored at −20°C. An RNA extraction kit, RNeasy Plus Mini Kit (QIAGEN, Hilden, Germany), was used to extract the RNA from the mouse tissue. Next, a cDNA synthesis kit, QuantiTect Reverse Transcription Kit (QIAGEN), was used to conduct RNA reverse transcription. Applied Biosystems TaqMan analysis kit and TaqMan Universal MasterMix II were used to conduct qPCR analysis. The following genes were analyzed: PPAR alpha (PPARα) (assay ID Mm00440939_m1), carnitine palmitoyltransferase 1 (CPT1, assay ID Mm01231183_m1), medium-chain acyl-CoA dehydrogenase (MCAD, assay ID Mm01323360_g1), sterol regulatory element-binding transcription factor 1 (SREBP-1, assay ID Mm00550338_m1), fatty acid synthase (FAS assay ID Mm00662319_m1), stearoyl-CoA desaturase-1 (SCD-1, assay ID Mm00772290_m1), sterol regulatory element-binding transcription factor 2 (SREBP-2, assay ID Mm01306292_m1), 3-hydroxy-3-methyl-glutaryl-CoA reductase (HMG-CoA reductase, assay ID Mm01282499_m1), and 3-hydroxy-3-methyl-glutaryl-CoA synthase (HMG-CoA synthase, assay ID Mm01304569_m1). Glyceraldehyde 3-phosphate dehydrogenase (GAPD; assay ID Mm99999915_g1) was used as the reference gene to calculate the relative amounts of gene transcriptions. The TaqMan reagents were purchased from Applied Biosystems, Foster City, California, USA.

### Statistical Analyses

Numerical data are displayed as mean ± standard deviation. One-way ANOVA was adopted, with the Tukey method used as the post hoc test. *p* < 0.05 indicates statistical significance.

## Results

### Pharmacodynamic Analysis of the Effect of GA on Mice With Diabetes Induced by HFD and STZ

#### Weight and Food Consumption Variations in the Normal, Diabetic, and Treatment Groups

**FIGURE 1:**
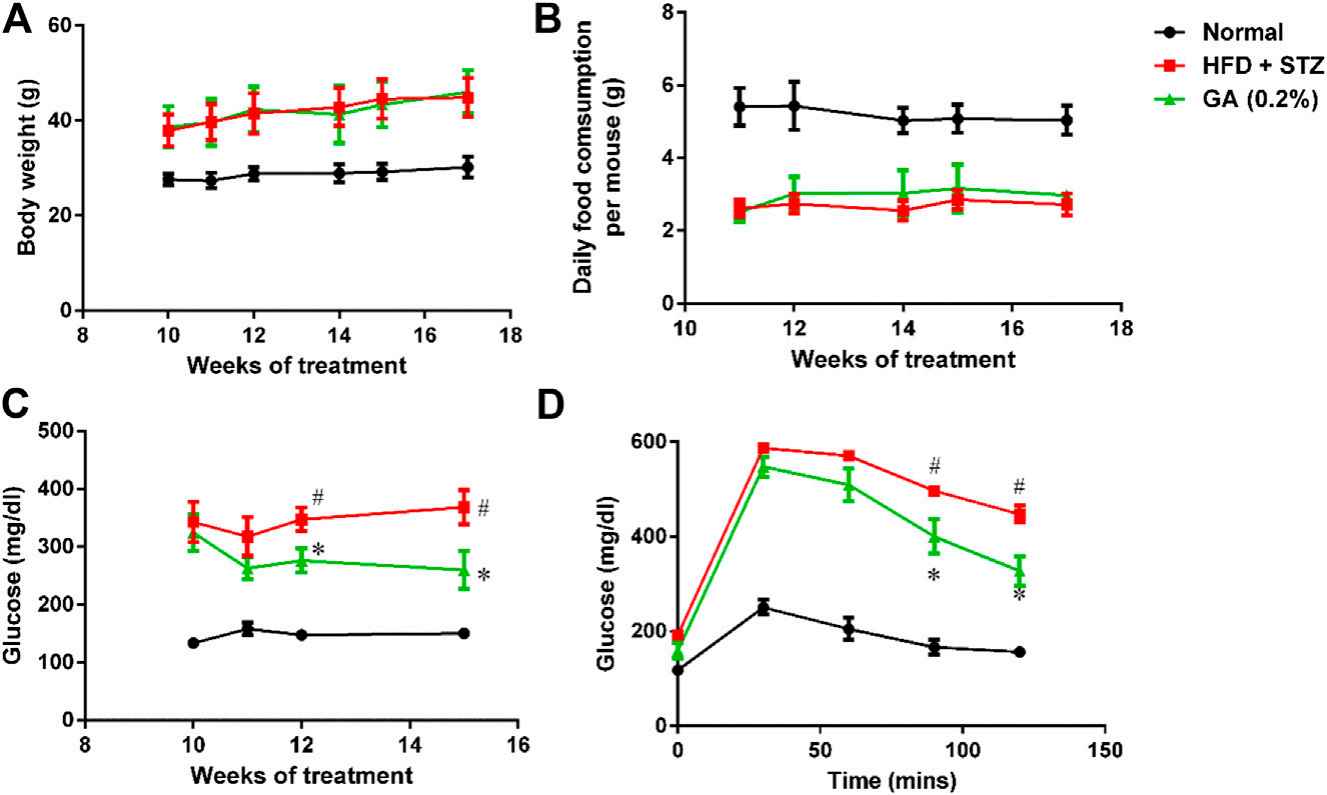
Analysis of physiological and biochemical parameters **(A)** Mice body weights **(B)** Variations in food consumption per mouse **(C)** Random blood glucose (RBS) test **(D)** Oral glucose tolerance test (OGTT). Seven to eight mice from each group were subjected to the RBS test on the 10th, 11th, 12th, and 15th week. Six mice from each group were subjected to the OGTT on the 16th week. In the OGTT, fasting glucose was conduct at 0 min; subsequently, the mice were orally fed with glucose (2 g/kg), and the blood glucose contents were measured at 30, 60, 90, and 120 min. # significant difference between the diabetic (HFD + STZ) and control (normal) groups (*p* < 0.05); * significant difference between the treatment (GA 0.2%) and diabetic groups (*p* < 0.05).

The weights of the diabetic group (HFD + STZ) and treatment group (GA 0.2%) showed significant increase than that of the normal group (Figure 1A). The GA treatment did not alleviate the obesity of the mice fed with the HFD (Figure 1A) or affect the food consumption (Figure 1B).

#### Random Blood Glucose (RBS) Test and Oral Glucose Tolerance Test (OGTT)

The RBS level of the diabetic group was significantly higher than that of the normal group (Figure 1C). The OGTT also revealed that the diabetic group experienced post-challenge hyperglycemia (Figure 1D). Postprandial blood glucose is affected by several factors, including the function of pancreatic β-cells and the sensitivity of related tissues and organs (muscles, fat, and the liver) to insulin. Therefore, these results confirmed that diabetes was successfully induced in the mice. After GA treatment, the RBS level of the mice on the 15th week was significantly lower than that of the mice in the diabetic group (Figure 1C). Moreover, the OGTT showed that GA facilitated alleviation of the post-challenge hyperglycemia in diabetic mice (Figure 1D). Thus, the aforementioned results indicate that GA alleviates high blood glucose.

#### Organ Weight and Serum Biochemical Values

**FIGURE 2:**
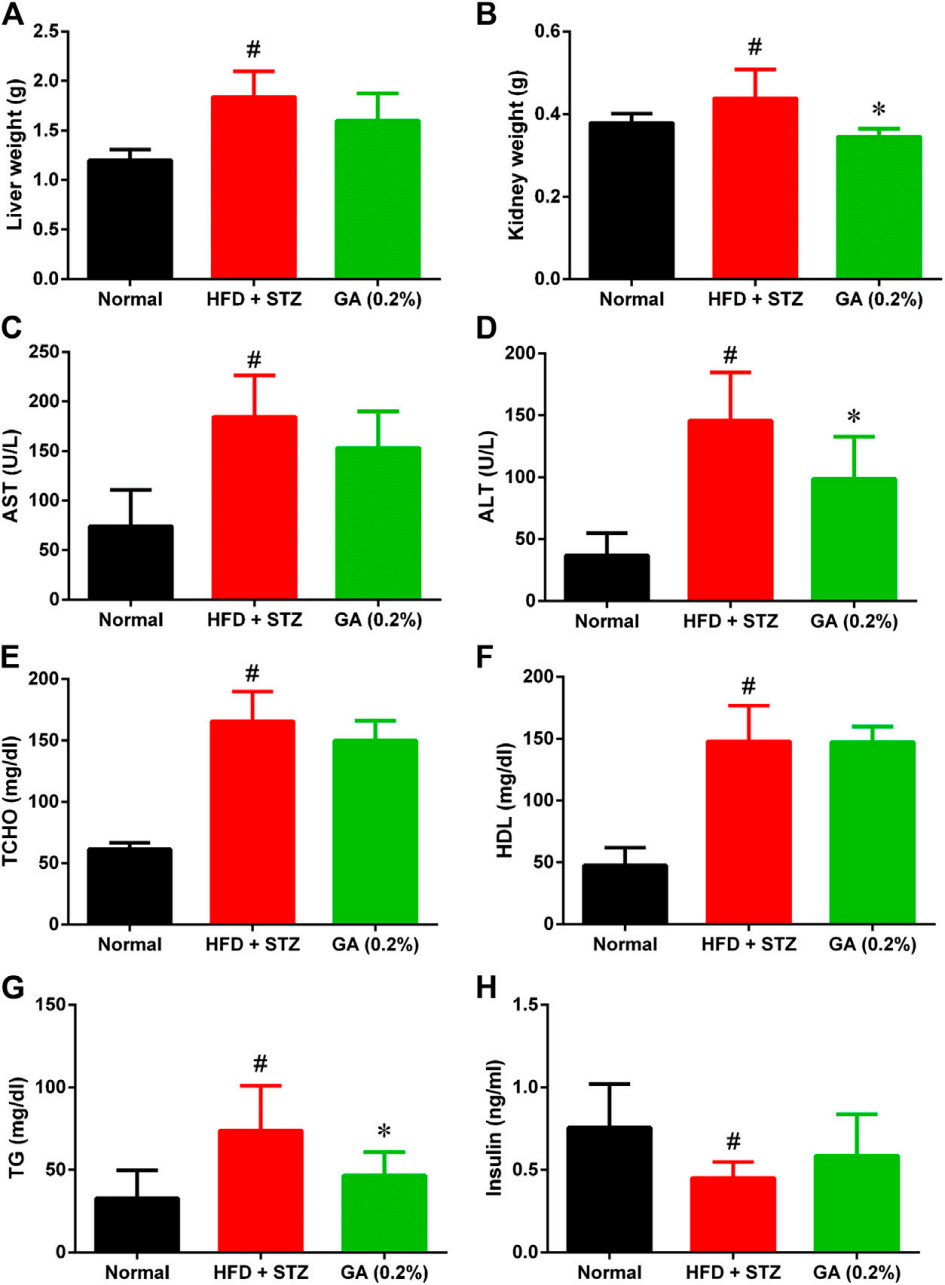
Organ weight and serum biochemical values of mice **(A)** Liver weights **(B)** Kidney weights **(C)** Aspartate transaminase (AST) **(D)** Alanine transaminase (ALT) **(E)** Total cholesterol (TCHO) **(F)** High-density lipoprotein (HDL) **(G)** Triglycerides (TG) **(H)** Insulin. # indicates significant difference between the diabetes group (HFD + STZ) and control (normal) group (*p* < 0.05); * indicates significant difference between the treatment group (GA 0.2%) and diabetes group (*p* < 0.05). Six to eight mice from each group were subjected to the AST and ALT test. Seven mice from the normal and diabetes groups were subjected to the insulin test, while five mice from the treatment group (GA 0.2%). Eight mice from each group were subjected to the remaining tests.

As shown in Figures 2A,B, the liver and kidney weights of the diabetic group were significantly higher than those of the normal group. However, the liver weight of the treatment group was not significantly lower, while the kidney weight of the treatment group was significantly lower than the diabetic group.

The serum biochemical values revealed that compared with the normal group, the diabetic group demonstrated significantly higher AST and ALT levels (Figures 2C,I), indicating that the liver function of the diabetic group was severely impaired. Moreover, the diabetic group also exhibited severe lipid metabolic disorders (Figures 2E–G); the TG, TCHO, and HDL levels of the diabetic group were significantly higher than those of the normal group. The liver function indices of the treatment group demonstrated a decreasing trend, and the ALT level was significantly lower than that in the diabetic group. GA treatment also alleviated the high TG of the mice with induced diabetes; however, their TCHO and HDL levels were not affected by the treatment. The insulin secretion of the diabetic group was significantly lower than that of the normal group (Figure 2H), indicating that the pancreatic β-cells of the diabetic group were severely damaged. The insulin secretion of the treatment group was not significantly higher than that of the diabetic group.

#### Histological Analysis of Liver, Muscle, and Adipose Tissues

**FIGURE 3:**
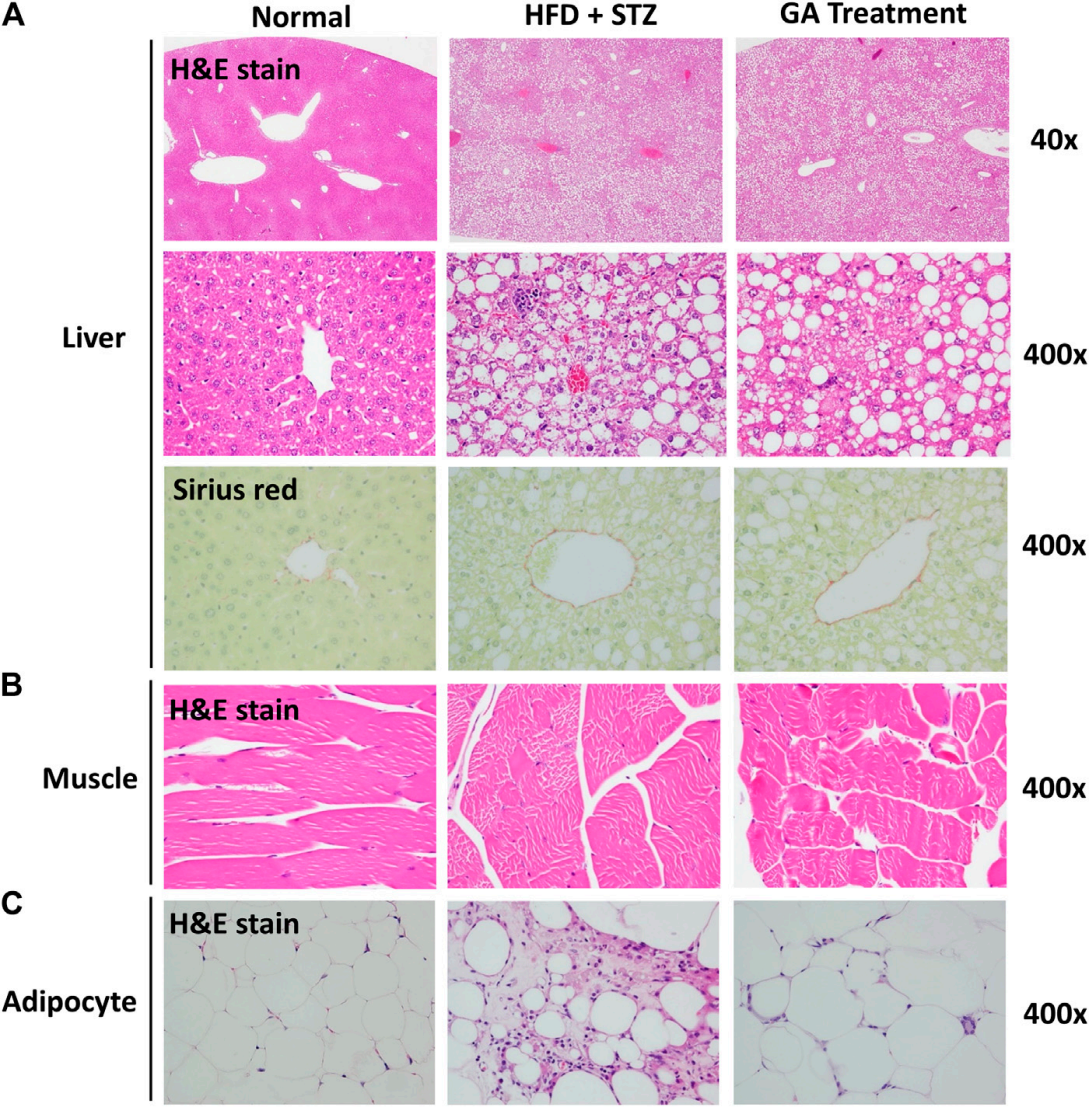
Histopathological analysis **(A)** Liver **(B)** Muscle **(C)** Adipose *(A)* is magnified by 40x and 400x *(B)* and *(C)* are magnified × 400.

No abnormalities were observed in the liver sections of the normal group (Figure 3A and Table 1), while diffuse hepatic steatosis, identified by primarily macrovesicular steatosis, was observed in the diabetic group. However, microvesicular steatosis was also observed in several regions of the section (Figure 3A and Table 1); this agrees with a previous study (Hebbard and George, 2011). Furthermore, swollen liver cells, ballooning degeneration, and focal necrosis were also observed in the section of the diabetic group, indicating inflammation (Hebbard and George, 2011). However, the Sirius red-dyed section of the diabetic group revealed no liver fibrosis (Figure 3A and Table 1).

**TABLE 1:** Pathological examination of liver.

	Liver	
	Microvesicular steatosis	Macrovesicular steatosis (ballooning degeneration)
Normal group	0.00	0.00
HFD + STZ group	5.00 ± 0.00	4.87 ± 0.33
GA treatment group	4.87 ± 0.33	4.37 ± 0.69

Lesion degree was graded from 1 to 5: 1 = minimal (<1%); 2 = slight (1–25%); 3 = moderate (26–50%); 4 = moderate/severe (51–75%); 5 = severe/high (76–100%). Values are presented as means ± SD (N = 8).

Hepatocellular ballooning is an essential indicator distinguishing the symptoms of NAFLD and NASH (Hebbard and George, 2011). It is noteworthy that the GA treatment alleviated the ballooning degeneration of the mice (Figure 3A). The data confirmed that the liver disease animal model induced through HFD and STZ had already progressed to NASH. Therefore, the results indicate that GA can delay the progression of NAFLD. GA exerted a weak effect on macrovesicular steatosis, slightly decreasing the synthesis and accumulation of large liver vacuoles (Figure 3A and Table 1). Thus, HFD and STZ can simultaneously induce NASH in a diabetes animal model, while GA can delay the progression of NASH.

Observation of the muscular tissue sections revealed no significant lesions in the normal or diabetic groups (Figure 3B). The adipose tissue sections showed that the adipose cells of the normal group were smaller than those of the other groups were and exhibited no noticeable pathological changes (Figure 3C). In contrast, the diabetic group demonstrated hypertrophy in the adipose cells along with fat necrosis and steatitis. Compared with the normal group, the treatment group exhibited unclear cellular boundaries and enlarged adipose cells and lipid droplets as well as macrophage infiltration; however, the extent of macrophage infiltrate was less than that in the diabetic group.

### Metabolomics Analysis on the Effect of GA on Mice With Diabetes Induced by HFD and STZ

#### Metabolite Identification

An NMR-based metabolomics platform was used to analyze endogenous metabolites in the blood, urine, liver, and muscle tissue samples of the normal, diabetic, and treatment groups. The signals of the endogenous metabolites displayed on NMR spectra were categorized (Dumas et al., 2006; Xu et al., 2012; An et al., 2013; Shi et al., 2013), and Chenomx 7.6 software was used to verifythem. The following metabolites were observed: serum (Supplementary Table S3), urine (Supplementary Table S4), water-soluble metabolites in the liver (Supplementary Table S5), lipid-soluble metabolites in the liver (Supplementary Table S6), and water-soluble metabolites in the muscles (Supplementary Table S7).

#### Multivariate Analysis

**FIGURE 4:**
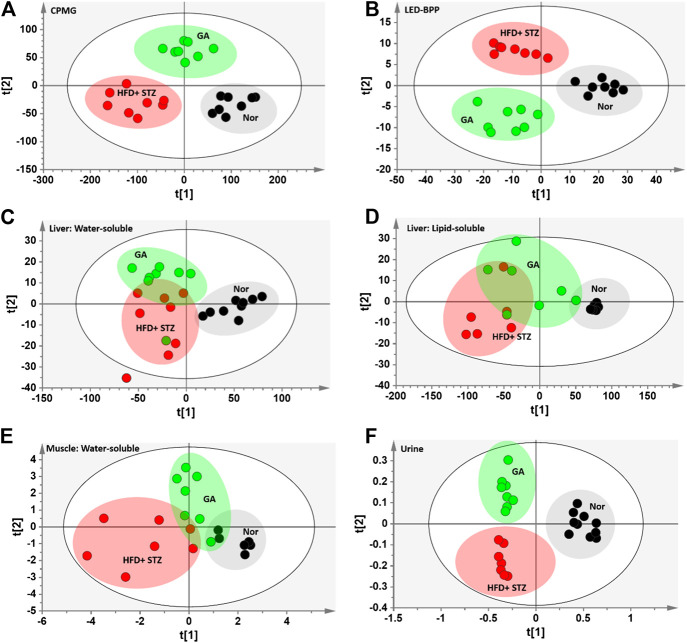
PLS-DA score plots **(A)** Carr-Purcell-Meiboom-Gill (CPMG) spectrum of serum samples for small molecule metabolites (R^2^X = 0.736, R^2^Y = 0.875, Q^2^ = 0.845) **(B)** Longitudinal eddy-current delay-bipolar pulse pair (LED-BPP) spectrum of serum samples for large-molecule metabolites (R^2^X = 0.841, R^2^Y = 0.892, Q^2^ = 0.874) **(C)** Nuclear Overhauser effect spectroscopy (NOESY) spectrum of liver samples for water-soluble metabolites (R^2^X = 0.736, R^2^Y = 0.586, Q^2^ = 0.359) **(D)** NOESY spectrum of liver samples for lipid-soluble metabolites (R^2^X = 0.927, R^2^Y = 0.567, Q^2^ = 0.332) **(E)** NOESY spectrum of muscle samples for water-soluble metabolites (R^2^X = 0.433, R_2_Y = 0.618, Q^2^ = 0.435) **(F)** NOESY spectrum of urine samples for metabolites (R^2^X = 0.646, R^2^Y = 0.892, Q^2^ = 0.799). Nor: normal group; HFD + STZ: diabetes group; GA: treatment group.

To compare the metabolism of the diabetic group with that of the normal and treatment groups, a partial least squares discriminant analysis (PLS-DA) with score plots was used to visualize the differences between the groups. As shown in the serum PLS-DA score plot, this metabolomics approach allows distinguishing between the effects induced by HFD and GA (Figures 4A,I). The score plots illustrate that from the T1 axis perspective, the diabetic group noticeably distinct from the normal group, indicating that the two groups exhibited dissimilar metabolic profiles, and that the diabetic group experienced severe metabolic disorders. However, the GA treatment did not markedly alleviate the disorders of water-soluble metabolites in the liver and urine (Figures 4C,I) but exerted a moderate effect on the disorders of lipid-soluble metabolites in the liver and water-soluble metabolites in the muscles (Figures 4D,E). It is noteworthy that the treatment group was distinct from the diabetic and normal groups, indicating that GA may exert an effect on multiple organs; however, it could only prevent some metabolic disorders. Therefore, the diabetic mice did not completely recover their health following GA treatment.

#### Lipid Changes in Serum and Liver

**FIGURE 5:**
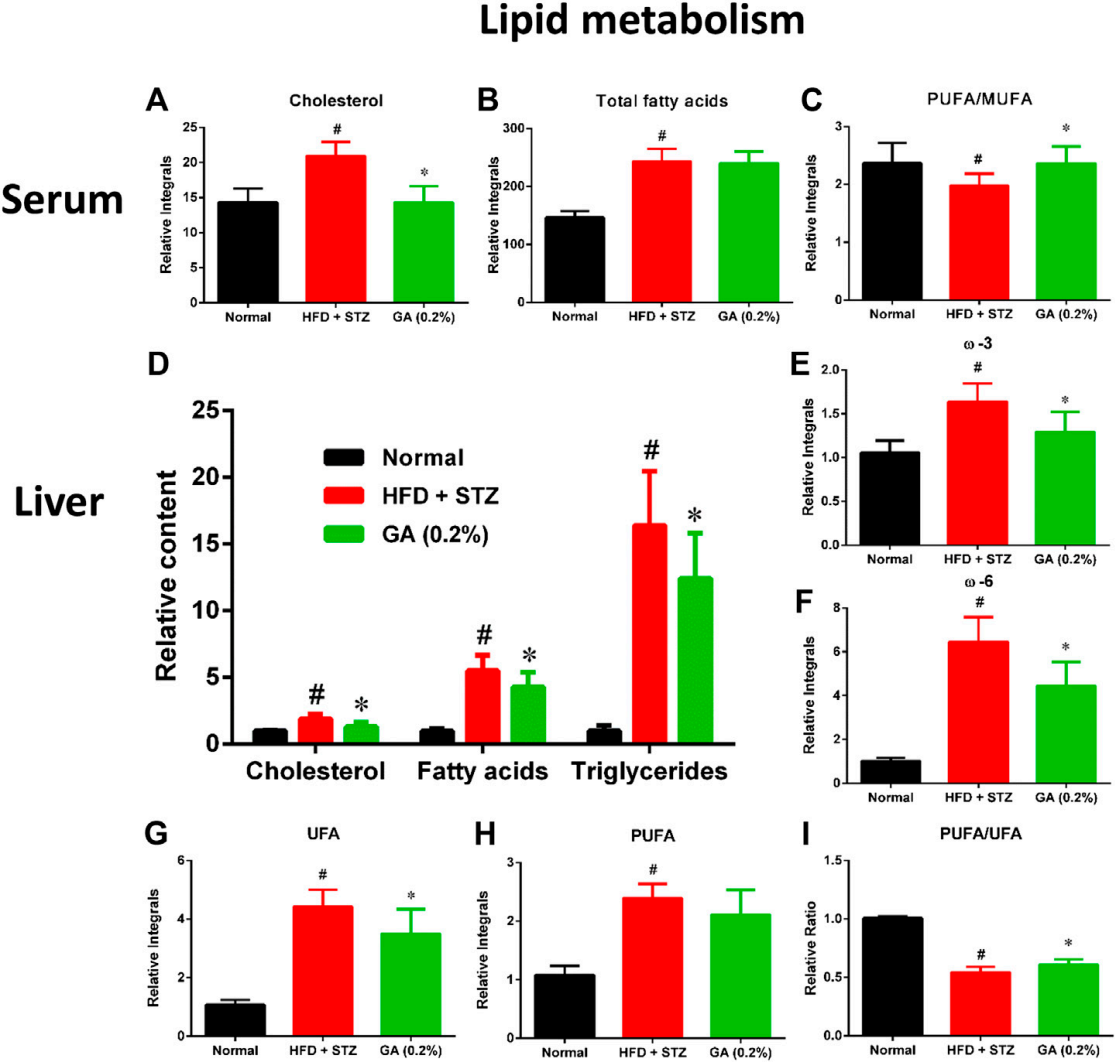
Effect of GA on serum and hepatic lipid metabolism **(A)** **–** **(C)**: serum lipids **(D)–(I)** hepatic lipids. # significant difference between the diabetic (HFD + STZ) and control (normal) groups (*p* < 0.05); * significant difference between the treatment (GA 0.2%) and diabetic groups (*p* < 0.05).

To further describe the differences between the metabolisms of the mouse groups, dissimilar metabolites were categorized according to biological functions to conduct semi-quantitative analysis. The results revealed severe metabolic disorders in the mice with diabetes induced by HFD and STZ (Figure 5). Specifically, the cholesterol and total fatty acid contents showed significant increase (Figures 5A,B). Moreover, the ratio of monounsaturated fatty acids to polyunsaturated fatty acids of the diabetic group decreased significantly (Figure 3C), indicating that the HFD + STZ treatment increased oxidative stress in mice (An et al., 2013). Alternatively, GA treatment alleviated the serum cholesterol metabolic disorder but not the accumulation of total fatty acids (Figures 5A,B). In addition, GA decreased oxidative stress in mice (Figure 5C).

Hepatic lipid analysis revealed that the liver lipid accumulation induced by HFD and STZ was primarily composed of TG, with a slight amount of cholesterol (Figure 5D). GA treatment decreased accumulation of hepatic lipids and alleviated hepatic steatosis, which concurred with the results of the histopathological sections (Figures 5E–H). The GA treatment partially prevented the accumulation of various lipids and alleviated the upregulated oxidative stress in the liver (Figure 5I).

#### Changes in Serum Metabolites

**FIGURE 6:**
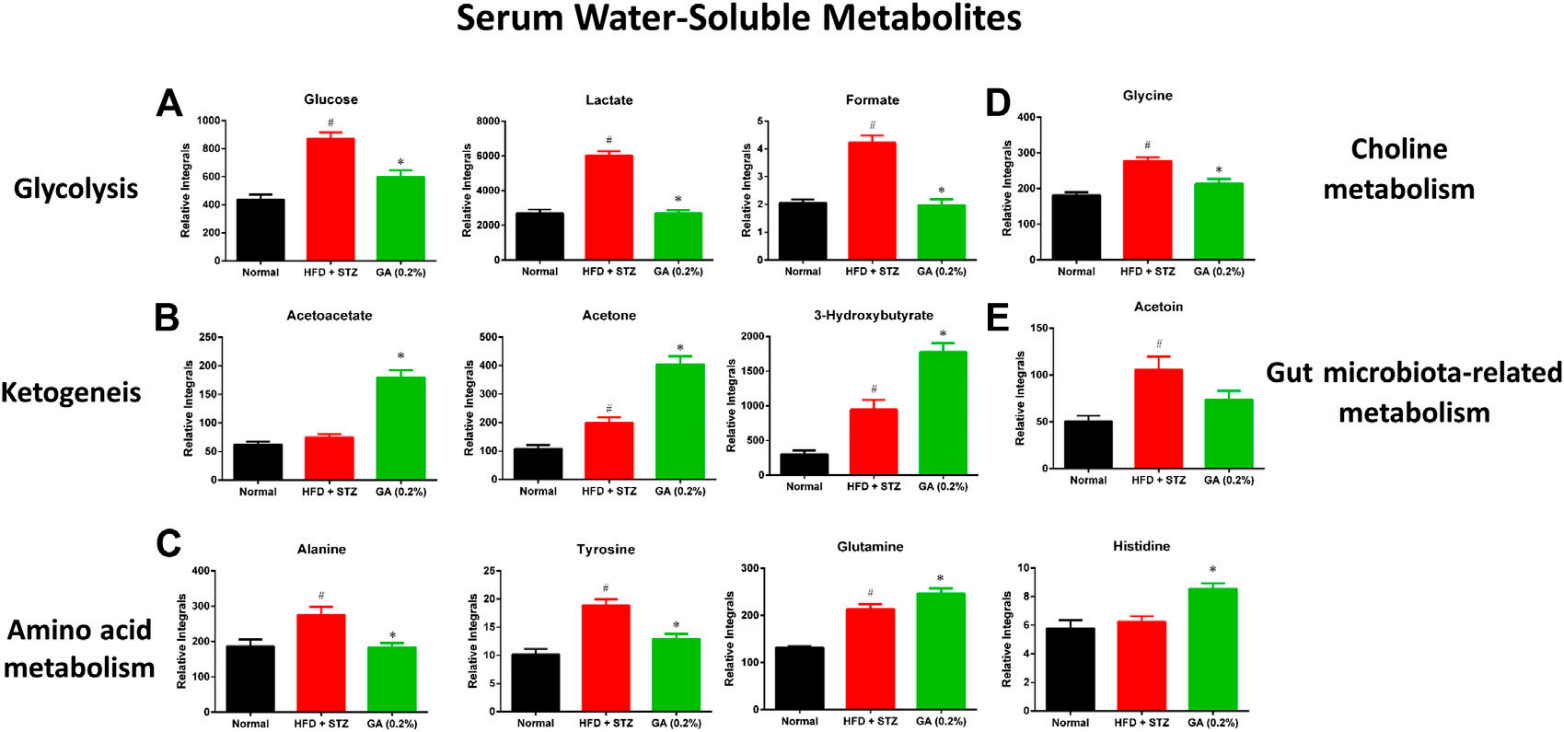
Effect of GA on serum metabolite changes **(A)** Glycolysis **(B)** ketogenesis **(C)** amino acid metabolism **(D)** choline metabolism **(E)** gut microbiota-related metabolism. # significant difference between the diabetic (HFD + STZ) and control (normal) groups (*p* < 0.05); * significant difference between the treatment (GA 0.2%) and diabetic groups (*p* < 0.05).

As shown in Supplementary Table S8, HFD + STZ treatment induced changes in metabolic pathways in the mice, including glycolysis, the tricarboxylic acid (TCA) cycle, ketogenesis, amino acid metabolism, choline metabolism, and gut microbiota-related metabolism. The serum samples reflected the average total changes in the metabolites of various tissues and organs. The results indicated that the HFD + STZ treatment induced a systemic effect, while GA treatment partially prevented some metabolic disorders and upregulated ketogenesis in the blood (Figure 6).

#### Changes in Urinary Metabolites

**FIGURE 7:**
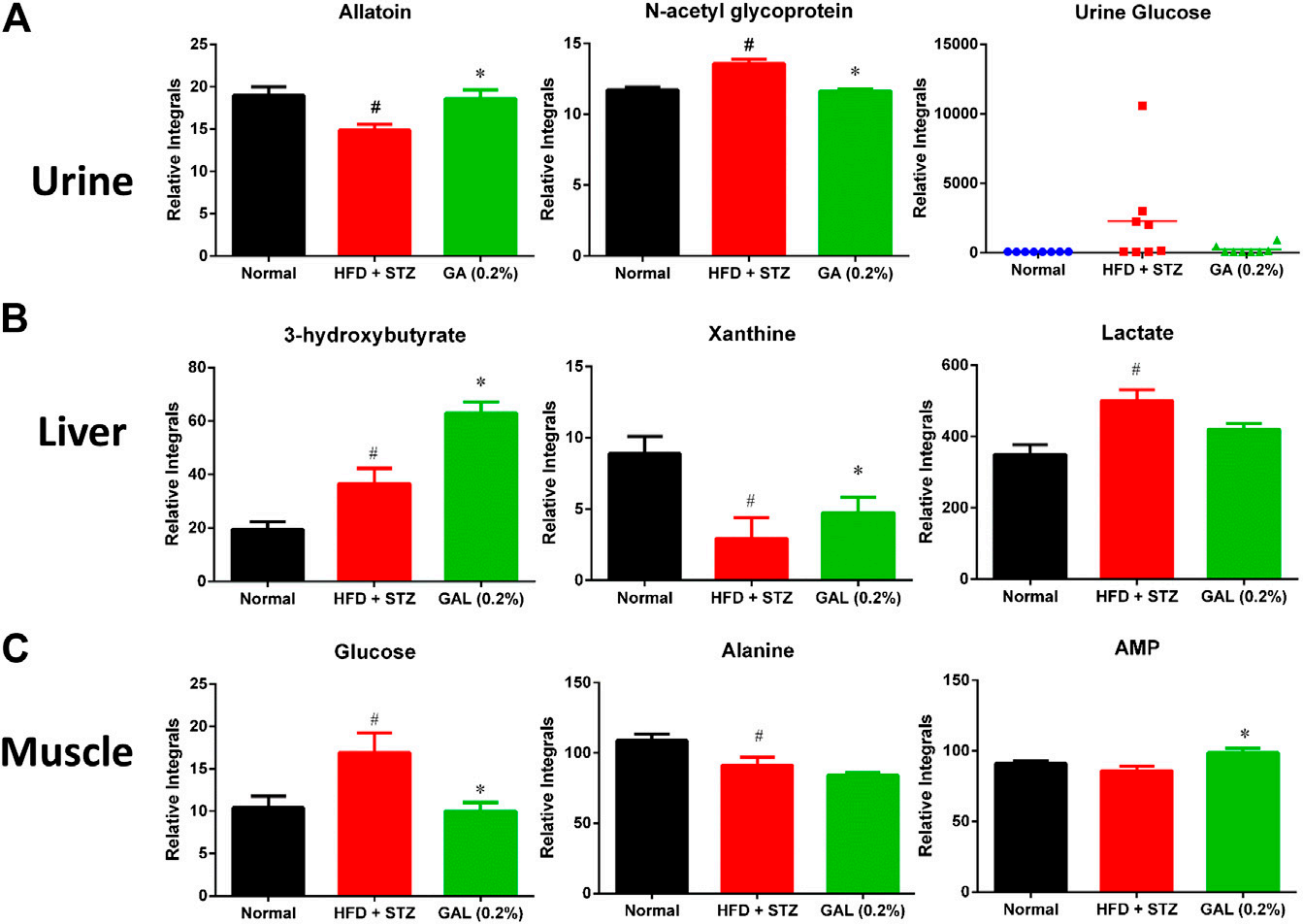
Effect of GA on changes in urinary, hepatic and muscle metabolites **(A)** Urine **(B)** Liver **(C)** muscle. # indicates a significant difference between the diabetes (HFD + STZ) and control (normal) groups (*p* < 0.05); * indicates a significance difference between the treatment (GA 0.2%) and diabetes groups (*p* < 0.05).

The HFD + STZ treatment induced changes in the urinary metabolites of various metabolic pathways, in particular metabolites involved in glycolysis, TCA cycle, creatine metabolism, amino acid metabolism, allantoin, and organic acids (Supplementary Table S9). The GA treatment prevented the downregulation of allantoin (Figure 7A) and the upregulation of urinary proteins and glucose in the mice induced with diabetes (Figure 7A). Hence, the GA treatment decreased the elevation of urinary protein and glucose levels.

#### Changes in Hepatic Metabolites

HFD + STZ treatment induced changes in the hepatic metabolism of the mice, including glycolysis and TCA cycles, ketogenesis, fatty acid metabolism, amino acid metabolism, purine metabolism, and choline metabolism (Supplementary Table S10); these changes showed similar trend as those observed for the serum metabolites. Compared with the diabetic group, the GA treatment group demonstrated increased levels of ketogenesis. Furthermore, the GA treatment prevented the metabolic disorder of xanthine (Figure 7B) and decreased the elevation of lactate (Figure 7B) yet failed to alleviate the disordered energy metabolism. These results concur with those of a previous study (Chao et al., 2014).

#### Changes in Muscle Metabolites

HFD + STZ treatment induced changes in only a few metabolites in the muscles of the mice (Supplementary Table S11), including the upregulation of glucose and inosine and downregulation of alanine. This indicates that HFD + STZ treatment exerted a small effect on the muscles of mice. The GA treatment decreased the glucose content in the muscular tissue of the mice as well as alleviated the downregulation of alanine; however, the extent of alleviation was not significant (Figure 7C). In addition, the GA treatment upregulated the adenosine monophosphate content in the muscular tissue of the mice with diabetes (Figure 7C).

### Gene Expression of Liver Lipid Metabolism

**FIGURE 8:**
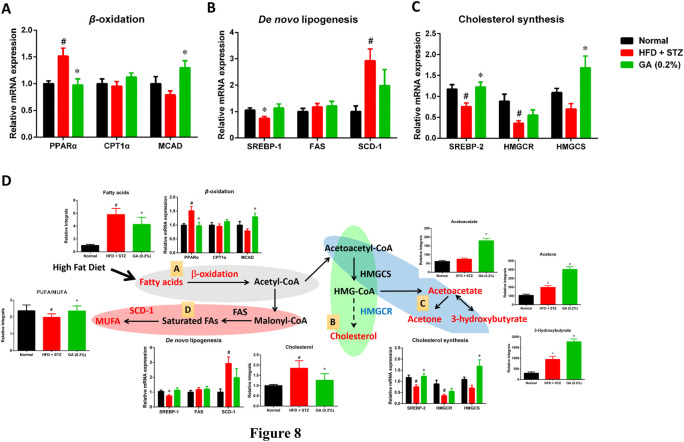
Possible mechanisms used by GA in alleviating accumulation of hepatic lipids in diabetic and NASH mice, induced by HFD and STZ **(A)** Regulation of *β*-oxidation genes **(B)** regulation of *de novo* lipogenesis genes; **(C)** regulation of cholesterol synthesis genes; **(D)** possible mechanisms, A Lipolysis—*β*-oxidation (gray); B cholesterol synthesis (green); C ketogenesis (blue); D lipid synthesis—*de novo* lipogenesis (red). Red indicates significant upregulation of metabolite or enzyme expression in diabetic mice, while blue indicates significant downregulation. Black indicates a metabolite or enzyme that is undetectable or exhibited non-significant changes. PPARα (peroxisome proliferator-activated receptor alpha), CPT1 (carnitine palmitoyltransferase, MCAD (medium-chain acyl-CoA dehydrogenase), SREBP-1 (sterol regulatory element-binding transcription factor 1), FAS (fatty acid synthase), SCD-1 (stearoyl-CoA desaturase-1), SREBP-2 (sterol regulatory element-binding transcription factor 2), HMG-CoA reductase (3-hydroxy-3-methyl-glutaryl-CoA reductase), HMG-CoA synthase (3-hydroxy-3-methyl-glutaryl-CoA synthase). Metabolites: MUFA (monounsaturated fatty acid), HMG-CoA (3-hydroxy-3-methylglutaryl-coenzyme A. # significant difference between the diabetic (HFD + STZ) and control (normal) groups (*p* < 0.05); * significant difference between the treatment (GA 0.2%) and diabetic groups (*p* < 0.05).

To investigate the differences between the gene expressions of liver lipid metabolites in the mouse groups, we analyzed the genes expressions of *β*-oxidation (Figure 8A), *de novo* lipogenesis (Figure 8B), and cholesterol synthesis (Figure 8C). The results revealed that the β-oxidation gene PPARα of the diabetic group was significantly upregulated, while the CPT1α and MCAD expressions were not significantly different from those of the normal group. The GA treatment prevented and significantly decreased the upregulated PPARα expression and increased the MCAD expression (Figure 8A). With respect to the *de novo* lipogenesis (Figure 8B), abundant gene expression of SCD-1 was observed in the diabetic group; this concurs with a previous study (Xie et al., 2010). The GA treatment downregulated the expression of SCD-1, but the extent of downregulation was not significant. For the cholesterol synthesis genes (Figure 8C), the expressions of SREBP-2 and HMGCR, in the liver of the mice with diabetes, were significantly downregulated. The GA treatment prevented the downregulation of SREBP-2 and upregulated the expression of HMGCS.

## Discussion

### GA Alleviates High Blood Glucose in an Animal Model

From a pathophysiological perspective, insulin resistance (relative insufficient insulin), and impaired insulin secretion (insufficient insulin) are essential to diabetes occurrence.

However, it is very interesting that only few muscle metabolites exhibited metabolic disorders (Supplementary Table S11), indicating that the mechanism of HFD and STZ in inducing high blood glucose in the diabetes animal model primarily involved damage of pancreatic *β*-cells for STZ, while the effect on the insulin resistance of muscle tissues was less severe. Examining the high insulin secretion in the serum sample of the treatment group revealed that the mechanism of GA to alleviate high blood glucose may be related to how GA relieves glucolipotoxicity and thus protects pancreatic *β*-cells from being damaged. Although the hyperglycemia caused by the model has less muscle involvement, it is still found that GA increased the content of adenosine monophosphate (AMP) in muscles, indicating that AMP-activated kinase (AMPK) may still be regulated in the process of lowering blood sugar (Doan et al., 2015). Whether GA decreases blood glucose by inducing AMP-activated kinase channels, requires further verification.

Previous studies have reported that GA can induce the expression of PPAR gamma (PPARγ) in the liver, muscles, and fatty tissues (Gandhi et al., 2014). The activity of PPARγ is related to insulin sensitivity. Therefore, we postulated that the mechanism by which GA alleviates high blood glucose may also be associated with the upregulation of PPARγ.

### GA Alleviates NAFLD in an Animal Model

Previous studies have asserted that high blood glucose is an essential factor that progresses the disease condition of obese mice to NASH (Lo et al., 2011). Here, the histopathological sections demonstrated evident ballooning degeneration and focal necrosis in the mice liver cells, indicating that the disease condition had already progressed to NASH (Hebbard and George, 2011). Noticeably, GA treatment alleviated this degeneration, implying that GA-mediated delay in the progression of NAFLD might be because of alleviation in the high blood glucose of the mice.

In addition, GA regulates the genes involved in the pathway and the changes in metabolites related to lipolysis (β-oxidation and ketogenesis), lipid synthesis (*de novo* lipogenesis), and cholesterol synthesis were examined. Accordingly, a hypothesis regarding the potential mechanisms of GA was proposed (Figure 8D).

(1) Ketone bodies are by-products generated through *β*-oxidation (Laffel, 1999). Compared with the diabetic group (HFD + STZ), the treatment group exhibited noticeable upregulation of ketone bodies, in particular acetone, acetoacetate, and 3-hydroxybutyrate, indicating the increased *β*-oxidation of fatty acids in the treatment group. GA treatment upregulated the gene expression of medium-chain acyl-coenzyme A (CoA) dehydrogenase, verifying the inference that the increase in ketone bodies after GA treatment might be related to GA inducing *β*-oxidation in the liver (Figure 8D).
(2) Increased *β*-oxidation increases the content of acetyl-CoA in the liver. Further analysis of the acetyl-CoA pathway revealed three plausible directions: *de novo* lipogenesis, cholesterol synthesis, or ketogenesis. Cholesterol synthesis first involves forming 3-hydroxy-3-methyl-glutaryl-CoA (HMG-CoA), followed by multiple steps for forming cholesterol. Moreover, HMG-CoA is an essential precursor of ketogenesis. Compared with the diabetic group, the treatment group demonstrated upregulated hydroxymethylglutaryl-CoA synthase expression (for catalyzing HMG-CoA synthesis), however, the expression of HMG-CoA reductase was not affected (for catalyzing cholesterol synthesis). In addition, the study results indicated that GA facilitates decreasing hepatic cholesterol and increasing ketone body formation. According to the aforementioned assertions, we inferred that GA treatment enables lipolysis, the products of which then undergo ketone metabolism instead of cholesterol synthesis (Figure 8D).
(3) In case of the protein expression associated with *de novo* lipogenesis in the diabetic group, only that of SCD-1 was affected, while the rate-limiting FAS was not. SCD-1 converts saturated fatty acids to monounsaturated fatty acids (MUFAs). The substantial upregulation of SCD-1 might be related to the downregulation of polyunsaturated fatty acid (PUFA)/MUFA ratio in the blood and liver of the diabetic group. GA administration inhibited the expression of SCD-1 and prevented the downregulation of the PUFA/MUFA ratio (Figure 8D).

The long-term metabolic changes observed in this study do not know whether the up-regulation of *β*-oxidation and ketogenesis is the cause of the improvement of fatty liver or the result of the improvement of fatty liver. However, according to the results of experimental design, there are differences between the normal group, diabetic group (HFD + STZ) and GA treatment group. Previous studies have indicated that genes related to fatty acid *β*-oxidation are up-regulated in HFD-induced obese mice. These genes are initially up-regulated (4 weeks) and then down-regulated (10 weeks) (Chan et al, 2008). In this study, HFD feeding lasted for 17 weeks. It is worth noting that in the GA group, the expression of MCAD increased significantly, which indicates that GA can upregulate genes related to *β*-oxidation. These results indicated that no compensatory adaptation occurred in response to HFD-induced short-term upregulation of genes related to fatty acid *β*-oxidation. The molecular mechanism of GA against *β*-oxidation needs to be further studied *in vitro*.

### HFD and STZ Induce Diabetes in an Animal Model and Cause Changes in Intestinal Microbiota Metabolism

Previous studies have indicated that the composition and quantity of intestinal microbiota in patients with obesity undergo substantial changes than in healthy people (Kumar et al., 2006); other studies have reported that the intestinal microbiota composition of mice with diabetes differs from nondiabetic mice, and that antibiotics can decrease the risk of diabetes (Brugman et al., 2006). These assertions indicate that changes in intestinal microbiota are crucial to the progression of metabolic diseases. This study confirmed the substantial upregulation of acetoin, which is related to pneumonia inducing *Klebsiella pneumoniae*, in the blood of the diabetic group. *K. pneumoniae* has been identified in the gastrointestinal tract and in patients with pyogenic liver abscesses (Fung et al., 2012), while urine metabolites related to intestinal microbiota metabolism, namely methylamine, hippurate, and trimethylamine, were downregulated. Hence, the intestinal microbiota in the diabetic mouse group may have undergone changes. Moreover, GA-rich fruits have reportedly facilitated the growth of probiotic bacteria in the intestine (Bialonska, et al., 2010). Here, we have confirmed that GA administration prevented the substantial upregulation of 2,3-butanediol, enabling serum acetoin to return to its regular level. However, GA was ineffective on urine metabolites, and its effect on balancing the growth of intestinal microbiota requires further verification.

### GA Alleviates Urine Protein and Urine Glucose in an Animal Model

The STZ-induced animal model used in this study is a typical diabetes and kidney disease model (Chow and Allen, 2015). This study verified that several mice in the diabetic group experienced severely high urine glucose. The kidney glucose absorption function of these mice may have been impaired; hence, loss of glucose through urination was detected, indicating that the kidneys of the mice may have also been damaged. The urine protein level of the diabetic group increased significantly, indicating that STZ-induced high blood glucose may have resulted in glomerular damage, thus generating additional urine protein, or that pathological change in the tubules may have prevented protein recycling. Urinary glucose was detected in only few mice in the treatment group, and their urine protein levels returned to those like the control group. These results indicate that GA treatment can potentially alleviate STZ-induced diabetes and kidney disease.

## Conclusion

Herein, we administered HFD and STZ to induce metabolic disorders in diabetic mice. The relevant metabolic pathways were examined to elucidate the mechanisms of metabolic disorders that may cause HFD and STZ-induced diabetes and NAFLD (Supplementary Figure S3). The results revealed that HFD and STZ-induced severe metabolic disorders in the diabetic mice, including metabolic disorders related to glucose, lipids, amino acids, purines, and pyrimidines as well as changes in intestinal microbiota. However, GA treatment alleviated the high blood glucose of the mice and decelerated the progression of NAFLD. This study is the first, to the best of our knowledge, to report the association between alleviation of lipid accumulation by GA and upregulation of *β*-oxidation and ketogenesis. The results of this study may supplement those of pharmacodynamics studies and facilitate characterization of novel GA mechanisms in alleviating metabolic diseases.

## Data Availability

The original contributions presented in the study are included in the article/Supplementary Material, further inquiries can be directed to the corresponding authors.
