# Supplementary material for: Gallic Acid Ameliorated Impaired Lipid Homeostasis in a Mouse Model of High-Fat Diet—and Streptozotocin-Induced NAFLD and Diabetes through Improvement of β-oxidation and Ketogenesis
Source: Front Pharmacol. 2021 Feb 12;11:606759. doi: 10.3389/fphar.2020.606759 (PMC7907449; doi:10.3389/fphar.2020.606759)
Supplement: Supplementary file 1 [file datasheet1.docx]

**Supplemental figure legends**

**Figure S1.** Animal experimental design for type 2 diabetes and NASH induced by HFD and STZ.

**Figure S2.** The initial and 9th-week weights of mice fed a normal diet or HFD # indicates a significant difference between the control and experimental groups (*p* < 0.05).

**Figure S3**. Possible mechanisms of GA in alleviating diabetes and NASH in mice with HFD + STZ-induced diabetes and NAFLD. Red indicates significant upregulation of a metabolite or enzyme in diabetic mice, while blue indicates significant downregulation. Black indicates a metabolite or enzyme that is undetectable or not significantly altered.

**Supplemental tables**

**Table S1.** Caloric composition of diet.

**Table S2.** NMR-based metabolomics analysis of different biological samples.

**Table S3.** Assignment of the NMR signals for the serum samples.

**Table S4.** Assignment of the NMR signals for the urine samples.

**Table S5.** Assignment of the NMR signals for the water-soluble metabolites in liver.

**Table S6.** Assignment of the NMR signals for the lipid-soluble metabolites in liver.

**Table S7.** Assignment of the NMR signals for the muscle metabolites.

**Table S8.** Semi-quantitative and statistical analysis on serum metabolites of related metabolic pathways.

**Table S9.** Semi-quantitative and statistical analysis on urine metabolites of related metabolic pathways.

**Table S10.** Semi-quantitative and statistical analysis on the water-soluble metabolites of related metabolic pathways observed in liver.

**Table S11.** Semi-quantitative and statistical analysis on the muscle metabolites of related metabolic pathways observed in muscle.

# **Supplemental Figures**

| 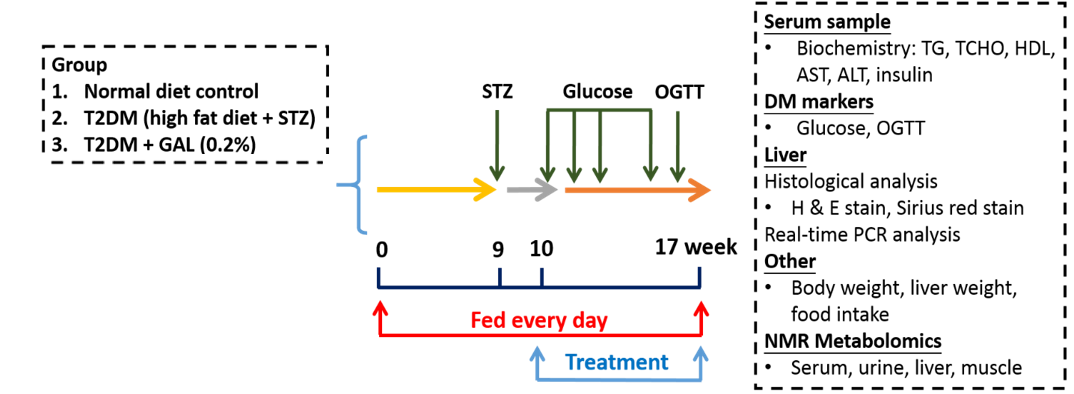 |
| --- |
| Figure S1 Animal experimental design for type 2 diabetes induced by HFD and STZ |

| 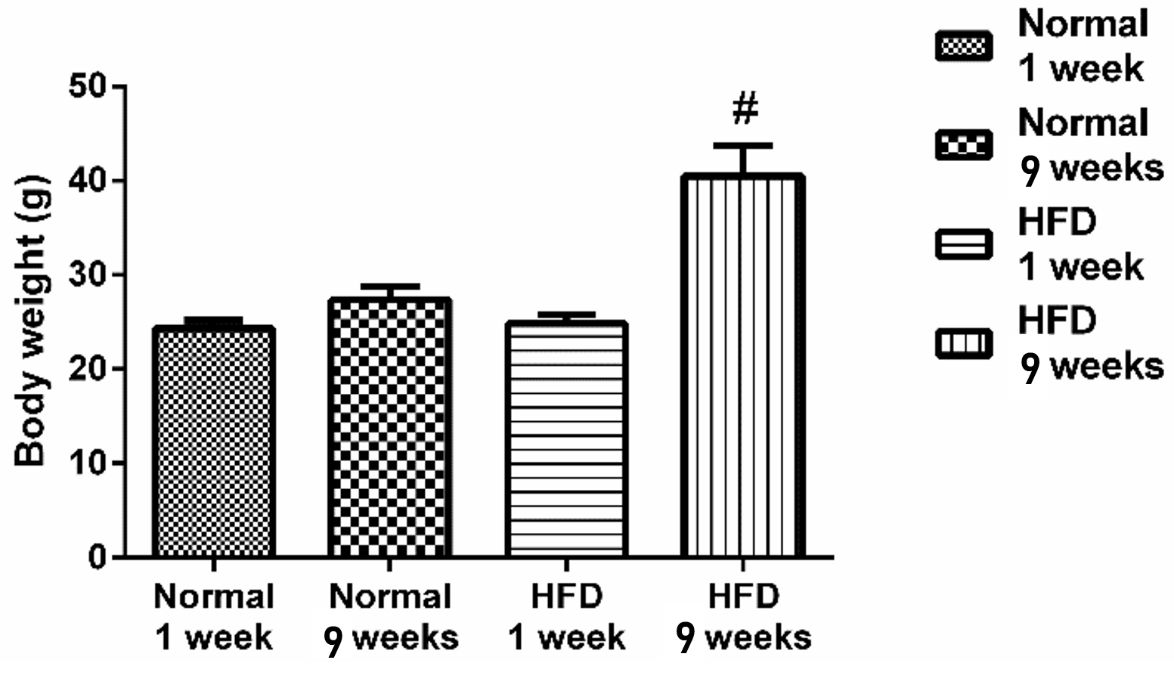 |
| --- |
| Figure S2 The initial and 9th-week weights of the mice fed with normal diet or HFD. # indicates significant difference between the control and experimental groups (p < 0.05). |


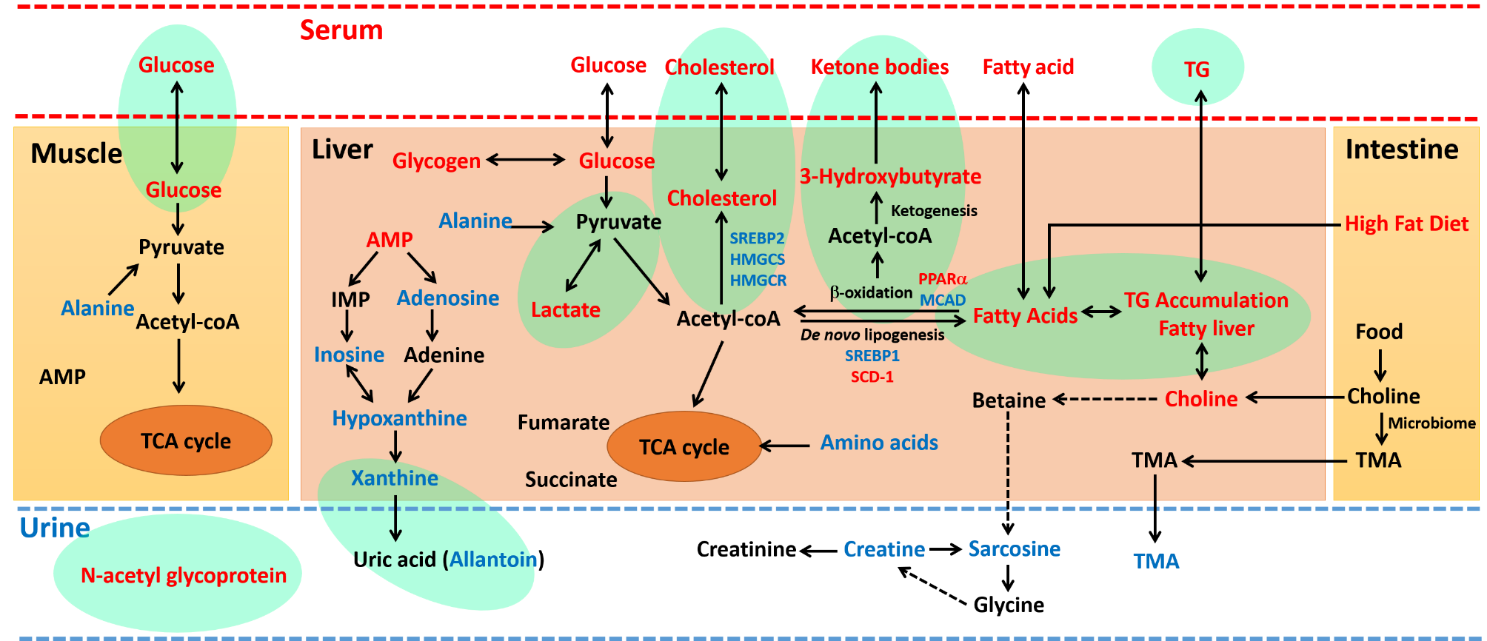


**Figure S3**. Possible mechanisms of GA in alleviating diabetes and NASH in mice with HFD + STZ-induced diabetes and NAFLD. Red indicates significant upregulation of a metabolite or enzyme in diabetic mice, while blue indicates significant downregulation. Black indicates a metabolite or enzyme that is undetectable or not significantly altered.

# **Supplemental Tables**

| **Table S1 Caloric composition of diet** | | | |
| --- | --- | --- | --- |
| **Diet** | **Carbohydrate (%)** | **Fat (%)** | **Protein (%)** |
| Normal diet  (4.14 kcal/gm) | 58.5 | 12.7 | 28.8 |
| High fat diet  (5.1 kcal/gm) | 20.3 | 61.6 | 18.1 |
|  | | | |

| **Table S2 NMR-based metabolomics analysis of different biological samples** | | | |
| --- | --- | --- | --- |
| **Samples** | **Solvent** | **Target metabolites** | **NMR pulse sequence** |
| Serum | D_2_O | Small molecule | CPMG  (Carr-Purcell-Meiboom-Gill) |
|  |  | Macromolecule | BPP-LED  (bipolar-pair longitudinal-eddy-current) |
| Urine | D_2_O | Small molecule / Macromolecule | NOESY |
| Tissues  (methanol−water phase) | D_2_O | Polar metabolites | NOESY |
| Tissues  (chloroform phase) | d-chloroform | Lipophilic metabolites | NOESY |

| **Table S3 Assignment of the NMR signals for the serum metabolites** | | | |
| --- | --- | --- | --- |
| **Code** | **Metabolites** | **Moieties** | ***δ* ^1^H (ppm) and multiplicity^a^** |
| 1 | Cholesterol  (LED-BPP) | R-C18-H_3_ | 0.68 (s) |
| 2 | Total FA  (LED-BPP) | R-CH_3_ | 0.88(t) |
| 3 | PUFA  (LED-BPP) | -CH=CH-CH_2_-(CH=CH-CH_2_-)_n_ | 2.75(m) |
| 4 | UFA  (LED-BPP) | -CH=CH- | 5.30(m) |
| 5 | Valine | γCH_3_  γ′CH_3_  βCH | 1.05(d)*  0.99(d)  2.28(m) |
| 6 | Isoleucine | γ′CH_3_  δCH_3_ | 1.01(d)  0.94(t) |
| 7 | Leucine | αCH  βCH_2_  γCH  δCH_3_  δ′CH_3_ | 3.75(d)  1.71(m)  1.69(m)  0.97(d)*  0.96(d)* |
| 8 | 3-Hydroxyisobutyrate | γCH_3_ | 1.06(d) * |
| 9 | 3-Hydroxybutyrate | γCH_3_  half αCH_2_  half αCH_2_ | 1.20(d) *  2.30(dd)  2.41(dd) |
| 10 | 2,3-Butanediol | β′CH_3_ | 1.14(d)* |
| 11 | Lactate | αCH  βCH_3_ | 4.11(q)  1.33(d)* |
| 12 | Threonine | αCH  βCH_2_  γCH_3_ | 3.60(d)  4.26(m)  1.33(d) |
| 13 | Lysine | αCH  εCH_2_  βCH  γCH_2_  δCH_2_ | 3.76(t)  3.03(t)*  1.92(m)  1.72(m)  1.45(m) |
| 14 | Alanine | αCH  βCH_3_ | 3.81(q)  1.48(d)* |
| 15 | Arginine | αCH  βCH_2_  γCH_2_  δCH_2_ | 3.77(m)  1.93(m)  1.69(m)  3.25(t) |
| 16 | Acetate | CH_3_ | 1.92(s)* |
| 17 | NAG  (N-acetyl glycoprotein) | CH_3_ | 2.04(s) |
| 18 | OAG  (O-acetyl glycoprotein) | CH_3_ | 2.14(s) |
| 19 | Glutamine | αCH  βCH_2_  γCH_2_ | 3.77(t)  2.14(m)  2.46(m)* |
| 20 | Glutamate | αCH  βCH_2_  γCH_2_ | 3.78(t)  2.06-2.13(m)  2.35(m) |
| 21 | Acetoacetate | CH_3_  CH_2_ | 2.27(s)*  3.44(s) |
| 22 | Acetone | CH_3_ | 2.23(s)* |
| 23 | Methionine | αCH  γCH_2_  δCH_2_ | 3.87(m)  2.65(t)  2.14(s) |
| 24 | Succinate | CH_2_ | 2.41(s) |
| 25 | Pyruvate | CH_3_ | 2.37(s)* |
| 26 | Proline | 4 CH_2_ | 3.34, 3.42 |
|  |  | 3 CH_2_ | 1.98 |
|  |  | 2 CH_2_ | 2.07, 2.35 |
|  |  | 1 CH | 4.14 |
| 27 | Citrate | half CH_2_  half CH_2_ | 2.54(d)*  2.66(d) |
| 28 | Asparagine | half βCH_2_  half βCH_2_  αCH | 2.87(dd)  2.95(dd)  4.00(dd) |
| 29 | Dimethylglycine | CH_3_  CH_2_ | 2.92(s)  3.72(s) |
| 30 | Creatine | CH_3_  CH_2_ | 3.04(s)  3.93(s) |
| 31 | Choline | N(CH_3_)_3_  NCH_2_  OCH_2_ | 3.21(s)*  3.52(m)  4.07(m) |
| 32 | Phosphorylcholine  (PC) | N(CH_3_)_3_  NCH_2_  OCH_2_ | 3.22(s)  3.60(m)  4.17(m) |
| 33 | Glycerophosphocholine  (GPC) | N(CH_3_)_3_  NCH_2_  OCH_2_ | 3.24(s)  3.70(m)  4.33(m) |
| 34 | Betaine | N(CH_3_)_3_  OCH_2_ | 3.27(s)  3.90(s) |
| 35 | Taurine | CH_2_SO_3_  CH_2_NH_2_ | 3.26(t)  3.44(t) |
| 36 | Scyllo-inositol | CH(ring) | 3.39(s) |
| 37 | 1,3-Dihydroxyacetone | CH_2_ | 4.405 |
| 38 | α-Glucose | 1-CH  2-CH  3-CH  4-CH  5-CH  6-CH_2_ | 5.24(d)*  3.56(dd)  3.73(dd)  3.42(dd)  3.83(dd)  3.83(dd) |
| 39 | β–Glucose | 1-CH  2-CH  3-CH  4-CH  5-CH  6-CH  6′-CH | 4.65(d)  3.27(dd)  3.50(dd)  3.40(dd)  3.47(dd)  3.74(dd)  3.90(dd) |
| 40 | Glycine | CH_2_ | 3.56(s)* |
| 41 | Urea | NH_2_ | 5.79(s)* |
| 42 | Fumarate | CH | 6.52(s) |
| 43 | Tyrosine | 3 or 5-CH  2 or 6-CH | 6.91(d)*  7.20(d) |
| 44 | Histidine | 4-CH  2-CH | 7.07(s)*  7.89(s) |
| 45 | Tryptophan | 4-CH  5-CH  6-CH  7-CH | 7.74(d)  7.21(t)  7.29(t)  7.55(d) |
| 46 | Phenylalanine | 2 or 6-CH  3 or 5-CH  4-CH | 7.32(dd)  7.43(dd)*  7.38(m) |
| 47 | Formate | CH | 8.46(s)* |

* Semi-quantitative peak

^a^ Peaks observed as singlet (s), doublet (d), triplet (t), quartet (q), multiple (m), or broad (b).

**Table S4 Assignment of the NMR signals for the urinary metabolites**

| **Code** | **Metabolites** | **Moieties** | ***δ* ^1^H (ppm) and multiplicity^a^** |
| --- | --- | --- | --- |
| 1 | Ethylmalonate | CH3  CH_2_  CH | 0.88(t)  1.73(m)  3.03(t) |
| 2 | Butyrate | CH3  αCH_2_  βCH_2_ | 0.89(t)  2.15(t)  1.60(m) |
| 3 | 2-Oxoisocaproate | δCH_3_  CH  γCH_2_ | 0.94(d)*  2.08(m)  2.62(m) |
| 4 | Valine | αCH  βCH  γCH_3_  γ′CH_3_ | 3.62(d)  2.28(m)  1.05(d)  0.99(d) |
| 5 | Methylmalonate | CH_3_  CH | 1.24(d)  3.17(q) |
| 6 | 3-Hydroxyisovalerate | CH_3_  CH_2_ | 1.26(s)*  2.38(s) |
| 7 | Lactate | αCH  βCH_3_ | 4.11(q)  1.33(d) |
| 8 | Threonine | αCH  βCH_2_  γCH_3_ | 3.60(d)  4.26(m)  1.33(d) |
| 9 | 2-Hydroxyisobutyrate | CH_3_ | 1.37(s)* |
| 10 | Alanine | βCH_3_ | 1.48(d) |
| 11 | Adipic acid | αCH_2_  βCH_2_ | 2.19(t)  1.54(m) |
| 12 | Acetate | CH_3_ | 1.92(s) |
| 13 | Acetamide | CH_3_ | 1.99(s) |
| 14 | NAG  (N-acetyl glycoprotein) | CH_3_ | 2.04(s) |
| 15 | Succinate | CH_2_ | 2.41(s)* |
| 16 | 2-Oxoglutarate  (Alpha-ketoglutaric acid) | αCH_2_  βCH_2_ | 2.44(t)*  3.01(t) |
| 17 | Citrate | half CH_2_  half CH_2_ | 2.53(d,16)*  2.67(d,16) |
| 18 | β-alanine | αCH_2_  βCH_2_ | 2.54(t)  3.21(t) |
| 19 | Methylamine | CH_3_ | 2.61(s) * |
| 20 | Sacrosine | CH_3_  CH_2_ | 2.72(s)  3.60(s)* |
| 21 | Trimethylamine (TMA) | CH_3_ | 2.89 (s) * |
| 22 | Dimethylglycine | CH_3_ | 2.93(s) |
| 23 | Creatine | CH_3_  CH_2_ | 3.04(s)*  3.93(s)* |
| 24 | Creatinine | CH_3_  CH_2_ | 3.05(s) *  4.06(s) * |
| 25 | Malonate | CH_2_ | 3.13(s) |
| 26 | Cis-aconitate | CH  CH_2_ | 5.71(s)  3.12(s) |
| 27 | N-Nitrosodimethylamine | CH_3_  CH_3_ | 3.16(s)  3.80(s) |
| 28 | Choline | N(CH_3_)_3_ | 3.21(s) * |
| 29 | Taurine | CH_2_SO_3_  CH_2_NH_2_ | 3.26(t)*  3.42(t) |
| 30 | Trimethylamine N-oxide  (TMAO) | CH_3_ | 3.27 (s) |
| 31 | Trans-aconitate | CH  CH_2_ | 6.59(s)*  3.45 (s) |
| 32 | Tartrate | CH, CH | 4.34(s)* |
| 33 | β–Glucose | 1-CH  2-CH  3-CH  4-CH  5-CH  6-CH  6′-CH | 4.45(d)  3.26(dd)  3.50(dd)  3.40(dd)  3.47(dd)  3.74(dd)  3.90(dd) |
| 34 | α-Glucose | 1-CH  2-CH  3-CH  4-CH  5-CH  6-CH_2_ | 5.24(d)  3.54(dd)  3.73(dd)  3.42(dd)  3.83(dd)  3.83(dd) |
| 35 | Allantoin | CH | 5.39(s)* |
| 36 | Urea | NH_2_ | 5.79(s) |
| 37 | Fumarate | CH | 6.52(s) |
| 38 | Tyrosine | 3 or 5-CH  2 or 6-CH | 6.89(d)  7.15(d) |
| 39 | 3-Indoxylsulfate | 5-CH  6-CH  2-CH  7-CH  4-CH | 7.21(t)  7.27(t)  7.35(s)  7.50(d)  7.71(d)* |
| 40 | Phenylacetate | 4-CH  3,5-CH  2,6-CH  CH_2_ | 7.278(t)  7.36(t)  7.28(t)  3.54(s) |
| 41 | N-Phenylacetylglycine | 3 or 5-CH  2 or 6-CH  4-CH  H_2_CCONH  H_2_CCOOH | 7.42(m)*  7.355(m)  7.36(m)  3.74(d)  3.66(s) |
| 42 | Uracil | O=CCH  CH | 7.5(d)  5.8(d) |
| 43 | Hippurate | 3 or 5-CH  4-CH  2 or 6-CH  αCH_2_ | 7.64(t)  7.83(d)*  7.55(t)  3.97(d) |
| 44 | Formate | CH | 8.46(s) |
| 45 | 4-PY  (N1-methyl-4-pyridone-3-carboxamide) | 3-CH  2-CH  6-CH  NCH_3_ | 6.70(d)  7.83(dd)  8.56(d) *  3.90(s) |
| 46 | Nicotinurate | 2-CH  4-CH  5-CH  6-CH  CH_2_ | 8.94(dd)  8.25(dd)  7.60(dd)  8.71(dd)  3.97(s) |
| 47 | Niacinamide | 4-CH  2-CH  5-CH  6-CH | 8.25 (dd)  8.94(dd)  7.60(dd)  8.71(dd) |
| 48 | Nicotinamide N-oxide | 5-CH  6-CH  4-CH  2--CH | 7.74 (dd)  8.09 (d)  8.49 (d)  8.74(t)* |
| 49 | Trigonelline | 2-CH  6 or 4-CH  5-CH  CH_3_ | 9.13(s)*  8.84(dd)  8.08(d)  4.44(s)* |
| 50 | 1-Methylnicotinamide | CH_3_  5-CH  4-CH  6-CH  2-CH | 4.47(s)  8.17(t)  8.90(d)  8.97(d)  9.28(s)* |

* Semi-quantitative peak

^a^ Peaks observed as singlet (s), doublet (d), triplet (t), quartet (q), multiple (m), or broad (b).

| **Table S5 Assignment of the NMR signals for the water-soluble metabolites in liver** | | | |
| --- | --- | --- | --- |
| **Code** | **Metabolites** | **Moieties** | ***δ* ^1^H (ppm) and multiplicity^a^** |
| 1 | Bile acids | CH_3_ | 0.73(s)* |
| 2 | Isoleucine | αCH  βCH  half γCH_2_  half γCH_2_  γ′CH_3_  δCH_3_ | 3.67(d)  1.99(m)  1.47(m)  1.27(m)  1.01(d)*  0.94(t) |
| 3 | Leucine | αCH  βCH_2_  γCH  δCH_3_  δ′CH_3_ | 3.75(d)  1.71(m)  1.69(m)  0.97(d)*  0.96(d) |
| 4 | Valine | αCH  βCH  γCH_3_  γ′CH_3_ | 3.62(d)  2.28(m)  1.05(d)*  0.99(d) |
| 5 | 3-Hydroxybutyrate | half αCH  half αCH  βCH_2_  γCH_3_ | 2.31(dd)  2.41(dd)  4.17(m)  1.20(d)* |
| 6 | 3-Aminoisobutyrate | β′CH_3_ | 1.18(d)* |
| 7 | Lactate | αCH  βCH_3_ | 4.11(q)  1.33(d)* |
| 8 | Lysine | αCH  εCH_2_  βCH  γCH_2_  δCH_2_ | 3.76(t)  3.03(t)  1.92(m)  1.72(m)  1.45(m) |
| 9 | Alanine | αCH  βCH_3_ | 3.78(q)  1.48(d)* |
| 10 | Arginine | αCH  βCH_2_  γCH_2_  δCH_2_ | 3.77(m)  1.93(m)  1.73(m)  3.25(t) |
| 11 | Acetate | CH_3_ | 1.92(s) |
| 12 | Glutamine  (2.44-2.48) | αCH  βCH_2_  γCH_2_ | 3.77(t)  2.14(m)  2.46(m)* |
| 13 | Glutamate  (2.33-2.36) | αCH  βCH_2_  γCH_2_ | 3.78(t)  2.06(m)  2.35(m)* |
| 14 | Pyruvate | CH_3_ | 2.37(s) |
| 15 | Methionine | αCH  βCH_2_  γCH_2_  δCH_2_ | 3.87(m)  2.16(t)  2.65(t)  2.14(s) |
| 16 | Succinate | CH_2_ | 2.41(s)* |
| 17 | Malate | half CH_2_  half CH_2_  αCH | 2.67(dd)  2.37(dd)  4.35(dd) |
| 18 | Sarcosine  (2.73-2.75) | CH_3_  CH_2_ | 2.74(s)*  3.65(s) |
| 19 | Trimethylamine | CH_3_ | 2.88(s) |
| 20 | Asparagine | half βCH_2_  half βCH_2_  αCH | 2.87(dd)  2.95(dd)  4.00(dd) |
| 21 | Dimethylamine | CH_3_ | 2.7 (s) |
| 22 | Dimethylglycine | CH_3_  CH_2_ | 2.93(s)  3.72(s) |
| 23 | Aspartate | βCH_2_  β′CH_2_  αCH | 2.81(dd)*  2.70(dd)  3.90(dd) |
| 24 | Glutathione  (GSH)  (2.95-3.0) | Gys *α*CH  Glu *α*CH  Gys βCH_2_  Glu γCH_2_  Glu βCH_2_ | 4.76(dd)  3.78(m)  2.98(dd)  2.52-2.57(m)  2.19(m) |
| 25 | Creatine | CH_3_  CH_2_ | 3.04(s)  3.93(s) |
| 26 | Creatinine | CH_3_  CH_2_ | 3.05(s)  4.06(s) |
| 27 | Choline | N(CH_3_)_3_  NCH_2_  OCH_2_ | 3.21(s)*  3.53(m)  4.07(m) |
| 28 | Carnitine | N(CH_3_)_3_ | 3.23(s)* |
| 29 | Taurine  (3.28-3.29) | CH_2_SO_3_  CH_2_NH_2_ | 3.27(t)*  3.42(t) |
| 30 | Methanol | CH_3_ | 3.36(s) |
| 31 | Ascorbate | CH  CHOH  CH_2_OH | 4.52(d)  4.0(m)  3.74(m) |
| 32 | Glycine | CH_2_ | 3.57(s)* |
| 33 | β-glucose | 1-CH  2-CH  3-CH  4-CH  5-CH  6-CH_2_ | 5.24(d)*  3.54(dd)  3.73(dd)  3.42(dd)  3.83(dd)  3.83(dd) |
| 34 | α–glucose | 1-CH  2-CH  3-CH  4-CH  5-CH  6-CH  6′-CH | 4.66(d)*  3.26(dd)  3.50(dd)  3.40(dd)  3.47(dd)  3.74(dd)  3.90(dd) |
| 35 | Fumarate | CH | 6.52(s)* |
| 36 | Tyrosine | 3 or 5-CH  2 or 6-CH  αCH | 6.91(d)*  7.20(d)  3.94(dd) |
| 37 | Histidine | 4-CH  2-CH | 7.12(s)*  7.89(s) |
| 38 | Tryptophan | 4-CH  5-CH  6-CH  7-CH | 7.74(d)  7.21(t)  7.28(t)  7.55(d) |
| 39 | phenylalanine | 2 or 6-CH  3 or 5-CH  4-CH | 7.33(dd)  7.43(dd)  7.38(m) |
| 40 | Uridine | 6-CH(ring)  5-CH(ring)  2-C'H(ribose)  1-C'H(ribose) | 7.89(d)*  5.91(d)  4.39(dd)  5.93(d) |
| 40 | Inosine | 2-CH  8-CH  2-CH′(ribose) | 8.32(s)*  8.22(s)  6.10(d) |
| 41 | Xanthine | 8-CH | 7.96(s)* |
| 42 | Hypoxanthine | 8-CH  2-CH | 8.21(s)  8.22(s)* |
| 44 | Formate | CH | 8.46(s)* |
| 45 | Nicotinamide adenine dinucleotide  (NAD+) | 2-CH  6-CH  4-CH  5-CH  2''-CH  8''-CH | 9.34(s)  9.13(d)  8.83  8.18(dd)  8.43(s)  8.12(s) |
| 46 | Adenosine monophosphate  (AMP) | 8-CH  2-CH  1-CH' | 8.59(s)*  8.25(s)  6.11(d) |
| 47 | Adenosine | 8-CH  2-CH  1-CH' | 8.36(s)*  8.25(s)  6.11(d) |
| 48 | ADP | 8-CH  2-CH  1-CH' | 8.52(s)  8.25(s)  6.11(d) |
| 49 | ATP | 8-CH  2-CH  1-CH' | 8.50(s)  8.25(s)  6.11(d) |
| 50 | GSSG | Glu-αCH  Glu-γCH_2_  Glu-βCH_2_  Glu-αCOOH  Cys-half CH  Cys-half CH  Cys-αCH | 3.78  2.55(m)  2.17(m)  3.0(dd)  3.32(dd)  4.76(t) |
| 51 | Creatinine | CH_3_  CH_2_ | 3.05(s)  4.06(s) |
| 52 | Nicotinurate | 2-CH  4-CH  5-CH  6-CH | 8.94(dd)*  8.27(dd)  7.60(dd)  8.72(dd) |

* Semi-quantitative peak

ADP: adenosine diphosphate; ATP: adenosine triphosphate; GSSG: glutathione disulfide.

^a^ Peaks observed as singlet (s), doublet (d), triplet (t), quartet (q), multiple (m), or broad (b).

Table S6 Assignment of the NMR signals for the lipid-soluble metabolites in liver

| **No.** | **Metabolites** | **Assignments** | **δ ^1^H (ppm), coupling constant, multiplicity ^a^** | **^13^C** |
| --- | --- | --- | --- | --- |
| **Cholesterol** | | | |  |
| 1 | Total Cholesterol | R-C18-H3  R-C26/27-H3  R-C21-H3 | 0.68 (s) ^*^  0.86 (2 x d) (one of the doublets is embedded in 0.88 triplet signal arising from fatty acid)  0.92 (d, *J* = 6.7) | 12.1  22.8  19.3 |
| 2 | Free Cholesterol | R-C19-H3 | 1.02 (s) | 19.5 |
| 3 | Esterified Cholesterol | R-C19-H3 | 1.04 (s) | 19.5 |
| **Glycerides** | | | |  |
| 4 | Triglycerides | Glycerol (C1-H) and Glycerol (C3-H)  Glycerol (C1-H’) and Glycerol (C3-H’)  Glycerol (C2-H) | 4.15 (d, *J* = 11.7, 5.9) ^*^  4.29 (d, *J* = 11.9, 4.4)  5.27 (m) | 62.8  62.3  68.7 |
| **Phospholipids** | | | |  |
| 5 | Total phospholipid  (except lysophosphatidylcholine) | Glycerol (C3-H_2_)  Glycerol (C2-H) | 3.97 (m) ^*^  5.21 (m) | 63.9  69.9 |
| 6 | Phosphatidylcholine (PC) | R-PO-CH_2_-CH_2_-N-(CH_3_)_3_  R-PO-CH_2_-CH_2_-N-(CH_3_)_3_  R-PO-CH_2_-CH_2_-N-(CH_3_)_3_ | 3.34 (s)  3.53 (m)  4.15 (m) | 54.3  71.6  62.6 |
| 7 | Glycerophosphocholine | R-PO-CH_2_-CH_2_-N-(CH_3_)_3_  R-PO-CH_2_-CH_2_-N-(CH_3_)_3_  R-PO-CH_2_-CH_2_-N-(CH_3_)_3_ | 3.34 (s)  3.72 (m)  4.35 (m) | 54.3  66.2  62.3 |
| 8 | Phosphatidylethanolamine (PE) | R-PO-CH_2_-CH_2_-N^+^H_3_  R-PO-CH_2_-CH_2_-N^+^H_3_ | 3.16 (s)  4.07 (m) | 40.9  32.3 |
| **Fatty acids** | | | |  |
| 9 | FA, (except ω-3) | FA chain C*H*_3_(CH_2_)_n_ | 0.88 (t, *J* = 6.6) ^*^, 0.89 (t, *J* = 6.0) ^*^ | 14.3 |
| 10 | FA, ω-3 (including DHA, EPA, and linolenic) | ω-3, C*H*_3_-CH_2_-C=C- | 0.97 (t, *J* = 7.2) ^*^ | 14.0 |
| 11 | FA, Total fatty acyl chains | FA chain -(C*H*_2_)_n_- | 1.30 (m) ^*^ | 29.6 |
| 12 | FA, βH_2_ | βH_2­­_, R-CH_2_CH_2_CO-OR | 1.61 (m) | 25 |
| 13 | FA | -CH=CH-CH_2_- | 2.03 (m) | 27.3 |
| 14 | FA, αH_2_ | αH_2_, -CH_2_CO-OR | 2.31 (m) | 34.0 |
| 15 | FA (including linoleic) | -CH=CH-CH_2_-(CH=CH-CH_2_-)_n_, n = 1 | 2.77 (t,, *J* = 6.6) ^*^ | 25.1 |
| 16 | FA (PUFA except linoleic) | -CH=CH-CH_2_-(CH=CH-CH_2_-)_n_, n ≥ 2 | 2.83 (m) ^*^ | 25.3 |
| 17 | FA | -CO-OCH_3_ | 3.67 (s) | 70.5 |
| 18 | FA (UFA, PUFA + MUFA) | -CH=CH- | 5.35 (m) ^*^ | 129.6 |

* Semi-quantitative peak. FA (fatty acid), DHA (docosahexaenoic acid), EPA (eicosapentaenoic acid and), UFA (unsaturated fatty acid), PUFA (polyunsaturated fatty acid), MUFA (monounsaturated fatty acid)

^a^ Peaks observed as singlet (s), doublet (d), triplet (t), quartet (q), multiple (m), or broad (b).

| Table S7 Assignment of the NMR signals for the muscle metabolites | | | |
| --- | --- | --- | --- |
| **Keys** | **Metabolites** | **Moieties** | ***δ*^1^H (ppm) and multiplicity** |
| 1 | Valine | αCH  βCH  γCH_3_  γ′CH_3_ | 3.62(d)  2.28(m)  1.05(d)*  0.99(d) |
| 2 | Isoleucine | αCH  βCH  half γCH_2_  half γCH_2_  γ′CH_3_  δCH_3_ | 3.67(d)  1.99(m)  1.47(m)  1.27(m)  1.01(d)  0.94(t) |
| 3 | Leucine | αCH  βCH_2_  γCH  δCH_3_  δ′CH_3_ | 3.75(d)  1.71(m)  1.69(m)  0.97(d)  0.96(d) |
| 4 | β–fucose | CH_3_  1-CH | 1.20(d)  5.25(d) |
| 5 | α–fucose | CH_3_  1-CH | 1.22(d)*  4.65(d) |
| 6 | Lactate | αCH  βCH_3_ | 4.11(q)  1.33(d)* |
| 7 | Alanine | αCH  βCH_3_ | 3.78(m)  1.48(d)* |
| 8 | Acetate | CH_3_ | 1.92(s) |
| 9 | Lysine | βCH  γCH_2_ | 1.92(m)  1.73(m) |
| 10 | Glutamine  (2.44-2.48) | αCH  βCH_2_  γCH_2_ | 3.77(t)  2.14(m)  2.46(m)* |
| 11 | Glutamate | αCH  βCH_2_  γCH_2_ | 3.78(t)  2.06-2.13(m)  2.35(m)* |
| 12 | Anserine | CH_3_  2-CH  4-CH | 3.76(s)  8.20(s)  7.11(s)* |
| 13 | Carnosine | 2-CH  4-CH  half βCH_2_  CH_2_  half βCH_2_  αCH  CH_2_ | 8.20(s)  7.08(s)*  3.24  3.21  3.02  4.47  2.72 |
| 14 | Methylamine | CH_3_ | 2.60(s) |
| 15 | Dimethylglycine  2.910-2.93 | CH_3_  CH_2_ | 2.92(s)  3.72(s) |
| 16 | Creatine | CH_3_  CH_2_ | 3.04(s)  3.93(s)* |
| 17 | Taurine | CH_2_SO_3_  CH_2_NH_2_ | 3.28(t)  3.43(t)* |
| 18 | Methanol | CH_3_ | 3.36(s) |
| 19 | α-glucose | 1-CH  2-CH  3-CH  4-CH  5-CH  6-CH_2_ | 5.24(d)  3.54(dd)  3.73(dd)  3.42(dd)  3.83(dd)  3.83(dd) |
| 20 | β–glucose  4.64-4.675 | 1-CH  2-CH  3-CH  4-CH  5-CH  6-CH  6′-CH | 4.65(d)*  3.26(dd)  3.50(dd)  3.40(dd)  3.47(dd)  3.74(dd)  3.90(dd) |
| 21 | Glycine | CH_2_ | 3.56(s)* |
| 22 | Adenosine monophosphate  (AMP) | 2-CH  7-CH  1-CH' | 8.59(s)*  8.25(s)  6.16(d) |
| 23 | ADP/ATP | 2-CH  8-CH  1-C'H(ribose) | 8.54(s)  8.27(s)  6.15(d) |
| 24 | NADPH/NADH | 2-CH  8-CH | 8.22(s)  8.50(s)  6.94(s)  6.1(d)  6.0(dd) |
| 25 | Fumarate | CH  COOH | 6.52(s) |
| 26 | Tyrosine | 3 or 5-CH  2 or 6-CH | 6.91(d)  7.20(d) |
| 27 | Phenylalanine | 2 or 6-CH  3 or 5-CH | 7.32(dd)  7.42(dd) |
| 28 | Formate | CH | 8.43(s) |
| 29 | Nicotinurate/niacinamide | 2-CH  4-CH  5-CH  6-CH | 8.94(dd)  8.27(dd)  7.60(dd)  8.72(dd) |
| 30 | Inosine | 2-CH  8-CH  2-CH′(ribose) | 8.35(s)*  8.22(s)  6.10(d) |

* Semi-quantitative peak

^a^ Peaks observed as singlet (s), doublet (d), triplet (t), quartet (q), multiple (m), or broad (b).

Table S8 Semi-quantitative and statistical analysis on serum metabolites of related metabolic pathways

| **Metabolites** | **Relative integral value ^a^** | | |
| --- | --- | --- | --- |
|  | **Normal** | **HFD + STZ** | **GA** |
| **Glycolysis and TCA cycle associated metabolites and intermediates** | | | |
| Glucose | 434.16 ± 34.30 | 872.26 ± 45.01^#^ | 599.00 ± 46.08* |
| Lactate | 2,698.31 ± 219.44 | 6,024.07 ± 245.16^#^ | 2,680.93 ± 203.09 |
| Pyruvate | 47.89 ± 1.92 | 55.81 ± 3.79 | 50.56 ± 2.2 |
| Formate | 2.06 ± 0.12 | 4.23 ± 0.27^#^ | 1.97 ± 0.21* |
| Citrate | 45.20 ± 5.17 | 83.06 ± 3.65^#^ | 80.09 ± 3.92 |
| Acetate | 64.81 ± 11.16 | 57.30 ± 6.63 | 66.98 ± 3.29 |
| **Ketogenesis and fatty acid metabolism** | | | |
| 3-Hydroxybutyrate | 302.69 ± 52.70 | 944.72 ± 142.44^#^ | 1,774.79 ± 126.2* |
| Acetoacetate | 61.55 ± 6.03 | 75.15 ± 5.09 | 179.41 ± 13.25* |
| Acetone | 107.85 ± 12.65 | 198.98 ± 19.48^#^ | 404.19 ± 28.91* |
| **Amino acids metabolism** | | | |
| Valine | 148.13 ± 6.90 | 141.81 ± 8.52 | 122.24 ± 7.28 |
| Leucine | 256.39 ± 11.55 | 280.96 ± 14.17 | 265.51 ± 19.17 |
| Alanine | 186.03 ± 20.58 | 275.11 ± 23.1^#^ | 183.42 ± 12.32* |
| Glutamine | 132.00 ± 2.06 | 213.4 ± 10.73^#^ | 246.24 ± 11.09* |
| Tyrosine | 10.16 ± 0.95 | 18.87 ± 1.07^#^ | 12.93 ± 0.91* |
| Histidine | 5.77 ± 0.59 | 6.26 ± 0.38 | 8.53 ± 0.40* |
| Phenylalanine | 14.00 ± 1.32 | 15.6 ± 2.07 | 11.09 ± 0.73 |
| Lysine | 64.09 ± 1.94 | 87.71 ± 1.56^#^ | 78.1 ± 5.29 |
| **Choline and choline associated metabolism** | |  | |
| Glycine | 181.09 ± 7.93 | 276.97 ± 10.95^#^ | 212.7 ± 14.21* |
| Creatine | 92.88 ± 3.79 | 130.66 ± 2.870^#^ | 132.08 ± 8.33 |
| **Urea cycle** | | | |
| Urea | 1.93 ± 0.40 | 0.89 ± 0.13^#^ | 0.92 ± 0.15 |
| **Gut microbiota-related metabolites** | | | |
| Acetoin | 50.76 ± 5.75 | 105.86 ± 13.66^#^ | 73.87 ± 9.37 |
| **Others** | | | |
| 2,3-Butanediol | 134.47 ± 6.98 | 233.09 ± 27.42^#^ | 140.1 ± 11.16* |

^a^The data are presented as the mean ± SEM. ^#^*p* < 0.05, versus normal diet mice; **p* < 0.05, versus high fat diet + STZ mice. TCA cycle: tricarboxylic acid-cycle

Table S9 Semi-quantitative and statistical analysis on the urinary metabolites of related metabolic pathways

| **Metabolites** | **Relative integral value x 1000^a^** | | | | | |
| --- | --- | --- | --- | --- | --- | --- |
|  | **Normal** | | **HFD + STZ** | | **GA** | |
| **Glycolysis and TCA cycle associated metabolites and intermediates** | | | | | | |
| Citrate | | 32.97 ± 3.8 | | 13.18 ± 2.22^#^ | | 8.40 ± 1.37 |
| Succinate | | 7.44 ± 0.49 | | 3.91 ± 0.21^#^ | | 4.25 ± 0.23 |
| 2-Oxoglutarate | | 9.34 ± 1.21 | | 4.51 ± 0.31^#^ | | 3.88 ± 0.28 |
| **Creatine metabolism** | | | | | | |
| Sarcosine | | 12.24 ± 0.37 | | 9.91 ± 1.33^#^ | | 9.34 ± 0.25 |
| Creatinine | | 22.28 ± 0.53 | | 20.96 ± 0.59 | | 23.42 ± 0.83 |
| Creatine | | 7.79 ± 0.43 | | 4.12 ± 0.34^#^ | | 4.53 ± 0.42 |
| **Gut microbiota-related metabolism** | | | | | | |
| Methylamine | | 3.31 ± 0.2 | | 1.37 ± 0.11^#^ | | 1.56 ± 0.07 |
| Hippurate | | 10.76 ± 0.39 | | 1.55 ± 0.13^#^ | | 1.72 ± 0.12 |
| Trimethylamine (TMA) | | 54.23 ± 2.28 | | 10.43 ± 2.22^#^ | | 18.29 ± 2.37 |
| **Nicotinate and nicotinamide metabolism** | | | | | | |
| Nicotinamide N-oxide | | 0.44 ± 0.03 | | 0.38 ± 0.04 | | 0.50 ± 0.03 |
| 1-Methylnicotinamide | | 0.63 ± 0.04 | | 0.64 ± 0.08 | | 0.66 ± 0.04 |
| Trigonelline | | 2.57 ± 0.13 | | 1.13 ± 0.11^#^ | | 0.95 ± 0.08 |
| 4-YP | | 0.64 ± 0.04 | | 0.79 ± 0.07 | | 0.89 ± 0.06 |
| **The metabolites of amino acids** | | | | | | |
| N-Phenylacetylglycine | | 4.70 ± 0.59 | | 7.12 ± 0.33^#^ | | 8.61 ± 0.30 |
| 3-Indoxylsulfate | | 1.02 ± 0.1 | | 0.62 ± 0.04^#^ | | 0.74 ± 0.05 |
| **Purine metabolism** | | | | | | |
| Allantoin | | 18.98 ± 1.02 | | 14.89 ± 0.66^#^ | | 18.6 ± 1.03* |
| **Organic acids and derivatives** | | | | | | |
| Trans-aconitate | | 0.93 ± 0.13 | | - | | - |
| Methylmalonate | | 7.74 ± 0.39 | | 7.32 ± 0.24 | | 8.08 ± 0.2 |
| 2-Oxoisocaproate | | 17.83 ± 0.34 | | 20.86 ± 0.64^#^ | | 19.69 ± 0.38 |
| 2-Hydroxyisobutyrate | | 4.47 ± 0.17 | | 4.84 ± 0.34 | | 4.95 ± 0.13 |
| **Others** | | | | | | |
| NAG | | 11.72 ± 0.2 | | 13.58 ± 0.31^#^ | | 11.63 ± 0.14* |
| Taurine | | 13.26 ± 1.19 | | 19.15 ± 2.27 | | 17.15 ± 2.18 |
| Tartrate | | 2.63 ± 0.04 | | 30.43 ± 2.42^#^ | | 26.43 ± 2.42 |

^a^The data are presented as the mean ± SEM. ^#^*p* < 0.05, versus normal diet mice; **p* < 0.05, versus high fat diet-fed mice. TCA cycle: tricarboxylic acid-cycle; NAG: N-acetyl glycoprotein; 4-YP: N1-methyl-4-pyridone-3-carboxamide

| Table S10 Semi-quantitative and statistical analysis on the water-soluble metabolites of related metabolic pathways observed in liver | | | | | |
| --- | --- | --- | --- | --- | --- |
| **Metabolites** | **Relative integral value ^a^** | | | | |
|  | **Normal** | | **HFD + STZ** | | **GA** |
| **Glycolysis and TCA cycle associated metabolites and intermediates** | | | | | |
| Glucose | 70.45 ± 8.56 | | 135.03 ± 23.77 | | 181.65 ± 15.42 |
| Lactate | 349.94 ± 26.87 | | 526.33 ± 19.18^#^ | | 420.15 ± 15.87* |
| Succinate | 28.82 ± 3.74 | | 39.76 ± 3.98^#^ | | 44.45 ± 3.38 |
| Fumarate | 5.14 ± 0.32 | | 2.81 ± 0.19^#^ | | 2.55 ± 0.21 |
| Formate | 1.86 ± 0.23 | | 1.68 ± 0.48 | | 2.19 ± 0.37 |
| **Ketogenesis and fatty acid metabolism** | | |  | | |
| 3-hydroxybutyrate | 19.47 ± 2.91 | | 36.71 ± 5.56^#^ | | 62.93 ± 4.28* |
| Carnitine | 55.6 ± 2.22 | | 37.14 ± 3.25^#^ | | 35.72 ± 2.51 |
| **Bile acid metabolism** | |  | |  | |
| Bile acid | 5.27 ± 1.27 | | 5.54 ± 0.59 | | 6.68 ± 0.61 |
| Taurine | 246.77 ± 15.68 | | 234.06 ± 14.64 | | 248.85 ± 8.41 |
| Glycine | 107.41 ± 5.71 | | 106.59 ± 6.58 | | 107.96 ± 5.66 |
| **Amino acids metabolism** | |  | |  | |
| Valine | 104.96 ± 9.34 | | 42.38 ± 3.65^#^ | | 45.02 ± 3.42 |
| Isoleucine | 35.43 ± 3.8 | | 12.58 ± 1.2^#^ | | 13.54 ± 1.24 |
| Leucine | 36.25 ± 3.91 | | 12.65 ± 1.35^#^ | | 13.27 ± 1.46 |
| Alanine | 187.82 ± 10.6 | | 123.62 ± 6.64^#^ | | 126.72 ± 7.32 |
| Glutamate | 59.57 ± 8.38 | | 29.22 ± 1.97^#^ | | 32.87 ± 1.45 |
| Glutamine | 20.46 ± 1.77 | | 65.32 ± 3.02^#^ | | 56.98 ± 1.88 |
| Aspartate | 8.33 ± 1.53 | | 3.18 ± 0.62^#^ | | 2.8 ± 0.41 |
| Tyrosine | 9.6 ± 0.83 | | 4.31 ± 0.46^#^ | | 4.2 ± 0.38 |
| Histidine | 6.81 ± 0.4 | | 3.5 ± 0.27^#^ | | 3.19 ± 0.32 |
| GSSG | 76.35 ± 3.28 | | 73.78 ± 1.94 | | 75.94 ± 1.57 |
| **Purine and pyrimidine metabolism** | | |  | | |
| AMP | 0.97 ± 0.18 | | 1.72 ± 0.31^#^ | | 1.74 ± 0.18 |
| Adenosine | 24.17 ± 1.56 | | 13.17 ± 1.75^#^ | | 14.86 ± 1.26 |
| Hypoxanthine | 5.7 ± 0.9 | | 2.98 ± 0.38^#^ | | 2.95 ± 0.24 |
| Xanthine | 8.88 ± 0.44 | | 2.94 ± 0.52^#^ | | 4.74 ± 0.4* |
| Uridine | 7.83 ± 0.69 | | 7.14 ± 0.63 | | 6.79 ± 0.39 |
| **Glycerophospholipid metabolism** | | |  | | |
| Choline | 64.93 ± 6.17 | | 34.65 ± 4.66^#^ | | 40.41 ± 2.85 |
| **Nicotinate and nicotinamide metabolism** | | |  | | |
| Nicotinurate | 6.56 ± 0.29 | | 4.54 ± 0.48^#^ | | 4.78 ± 0.44 |
| ^a^The data are presented as the mean ± SEM. ^#^*p* < 0.05, versus normal diet mice; **p* < 0.05, versus high fat diet-fed mice. TCA cycle: tricarboxylic acid-cycle | | | | | |

Table S11 Semi-quantitative and statistical analysis on the muscle metabolites of related metabolic pathways observed in muscle

| **Metabolites** | **Relative integral value ^a^** | | |
| --- | --- | --- | --- |
|  | **Normal** | **HFD + STZ** | **GA** |
| **Glycolysis and TCA cycle associated metabolites and intermediates** | | | |
| Glucose | 10.4 ± 1.36 | 16.9 ± 2.33^#^ | 10.02 ± 1.02* |
| Lactate | 1,538.67 ± 153.54 | 1,656.58 ± 76.84 | 1,311.48 ± 142.66 |
| Acetate | 8.64 ± 0.74 | 8.71 ± 0.64 | 9.27 ± 0.48 |
| **Amino acids metabolism** | | | |
| Valine | 12.92 ± 2.24 | 11.99 ± 0.79 | 10.44 ± 1.89 |
| Alanine | 109 ± 4.43 | 91.42 ± 5.48^#^ | 84.03 ± 2.07 |
| Glutamine | 22.8 ± 2.32 | 33.85 ± 4.91 | 36.24 ± 3.14 |
| Glutamate | 12.53 ± 3.86 | 16.23 ± 3.63 | 19.74 ± 1.93 |
| Glycine | 60.6 ± 4.89 | 65.39 ± 4.79 | 53.56 ± 1.34 |
| **Histidine metabolism** | | | |
| Carnosine | 118.9 ± 6.49 | 100.3 ± 6.44 | 109.58 ± 3.17 |
| Anserine | 42.07 ± 2.42 | 32.4 ± 3.01 | 34.94 ± 1.92 |
| **Creatine metabolism** | | | |
| Creatine | 921.21 ± 10.26 | 898.75 ± 15.77 | 977.03 ± 15.04 |
| **Purine metabolism** | | | |
| AMP | 91.02 ± 1.91 | 86.09 ± 2.86 | 98.52 ± 3.29* |
| Inosine | 6.63 ± 0.27 | 8.84 ± 0.54^#^ | 8.07 ± 0.53 |
| **Others** | | | |
| Fucose | 11.12 ± 1.65 | 15.03 ± 0.69 | 16.46 ± 2.15 |
| Taurine | 1,304.6 ± 19.81 | 1,366.32 ± 29.56 | 1381.02 ± 20.25 |

^a^The data are presented as the mean ± SEM. ^#^*p* < 0.05, versus normal diet mice; **p* < 0.05, versus high fat diet-fed mice. TCA cycle: tricarboxylic acid-cycle
